# A New Approach for Effective Retrieval of Medical Images: A Step towards Computer-Assisted Diagnosis

**DOI:** 10.3390/jimaging10090210

**Published:** 2024-08-26

**Authors:** Suchita Sharma, Ashutosh Aggarwal

**Affiliations:** Computer Science and Engineering Department, Thapar Institute of Engineering and Technology, Patiala 147004, Punjab, India; ssharma60_phd20@thapar.edu

**Keywords:** CBMIR systems, computer-assisted diagnosis, feature extraction, medical imaging, texture information

## Abstract

The biomedical imaging field has grown enormously in the past decade. In the era of digitization, the demand for computer-assisted diagnosis is increasing day by day. The COVID-19 pandemic further emphasized how retrieving meaningful information from medical repositories can aid in improving the quality of patient’s diagnosis. Therefore, content-based retrieval of medical images has a very prominent role in fulfilling our ultimate goal of developing automated computer-assisted diagnosis systems. Therefore, this paper presents a content-based medical image retrieval system that extracts multi-resolution, noise-resistant, rotation-invariant texture features in the form of a novel pattern descriptor, i.e., MsNrRiTxP, from medical images. In the proposed approach, the input medical image is initially decomposed into three neutrosophic images on its transformation into the neutrosophic domain. Afterwards, three distinct pattern descriptors, i.e., MsTrP, NrTxP, and RiTxP, are derived at multiple scales from the three neutrosophic images. The proposed MsNrRiTxP pattern descriptor is obtained by scale-wise concatenation of the joint histograms of MsTrP×RiTxP and NrTxP×RiTxP. To demonstrate the efficacy of the proposed system, medical images of different modalities, i.e., CT and MRI, from four test datasets are considered in our experimental setup. The retrieval performance of the proposed approach is exhaustively compared with several existing, recent, and state-of-the-art local binary pattern-based variants. The retrieval rates obtained by the proposed approach for the noise-free and noisy variants of the test datasets are observed to be substantially higher than the compared ones.

## 1. Introduction

### 1.1. Background and Motivation

With the advent of the digital age, there has been a rapid escalation in the use of digital images in applications like medical diagnosis. This has led to the formation of numerous digital image repositories and image archives. The use of biomedical images has enormously helped doctors make accurate diagnoses for patients [1]. Another aspect associated with the use of digital images is the ever-increasing demand for automated computer-assisted diagnosis (CAD) using machine learning [2]. After the COVID-19 pandemic severely affected the entire world, a lot of work has been put towards the computer-assisted detection of corona virus in patients’ chest X-ray images or CT images. This has further fueled the demand for CAD systems to ease the burden on already burdened and scarce medical personnel. Thus, our presented work focuses on one such branch of CAD systems, i.e., medical image retrieval (MIR) systems [3]. With the formation of medical image repositories and archives, effective data management and retrieval are required to ensure their optimum usage [4]. The sole purpose of MIR systems is to retrieve the most relevant and meaningful images in response to a user’s query from the existing database. MIR systems can be categorized based on the way in which the user submits their query, i.e., text-based image retrieval or content-based image retrieval [5].

In text-based image retrieval, the user submits their query in the form of a string containing keywords like lung, brain, tumor, etc., which are used to search relevant images on the basis of automatic or manual annotation of the image. Manual annotation is subjective and infeasible on large datasets, leading to its inability to describe complex visual properties (such as irregular shapes, varying textures) contained within medical images, thereby posing significant challenges for their retrieval [6]. A traditional patient diagnosis includes a comprehensive examination of the patient’s data (both image and non-image) in conjunction with the doctor’s previous encounters with similar situations. It has been observed that knowledge from similar cases has been greatly enhanced with the use of the visual content of medical images [1,2]. Therefore, the capability to search based on medical image information is growing in significance. Content-based medical image retrieval (CBMIR) retrieves images by extracting relevant visual properties such as shape, color, and texture. CBMIR systems can assist in diagnosis prognosis by retrieving images of the same anatomic location affected by the same disease [4]. Using a CBMIR system, a clinician can search a database of known instances for images with similar traits to those found in the abnormal diagnostic image. CBMIR offers pertinent supporting information from previous instances, presenting the physician with training examples with a proven diagnostic record, enabling them to garner confidence in their prognosis of the detected disease. Less experienced practitioners can benefit from the expertise by using visually identical retrieved images as a form of expert consultation. CBMIR would be beneficial for medical students and researchers to search and explore extensive collections of disease-related images based on their visual characteristics, serving as a valuable training tool. CBMIR’s success will lead to advancements in medical services and research, including disease tracking, differential diagnosis, noninvasive surgical planning, clinical training, etc. [5].

### 1.2. State of the Art

A lot of effort has been put forth by researchers across the globe in developing such CBMIR systems, employing a wide range of feature extraction strategies, mainly harnessing the texture information from medical images. Medical images (mostly gray-scale images) are rich in texture information. Therefore, their examination usually requires interpretation of tissue appearance, i.e., local intensity variations based on different texture properties such as smoothness, coarseness, regularity, and homogeneity [1]. Since texture information holds such a huge importance, texture-based feature extraction methods have become one of the most widely used techniques for medical image analysis, classification, and retrieval [7]. All different forms of feature descriptors (color, texture, and shape) can further be categorized into local feature descriptors (LFDs) and global feature descriptors (GFDs) [8]. GFDs capture the overall aspect of an image, such as information corresponding to shape and structure, e.g., information, etc., whereas LFDs capture localized information of an image such as the presence of a lesion in a particular location that is very small in size with respect to the entire image. GFDs are not able to accurately represent information about such lesions. In LFDs, an image is divided into sub-images and the final feature vector of the entire image is formed by appending the information extracted from each of the sub-images. In GFDs, the resultant feature vector is obtained by using all the pixels together as a whole [9]. Among the LFDs, local binary pattern (LBP) [10] has been most widely adopted for extracting texture information. The computation of LBP involves the encoding of pixel intensity differences within a local neighborhood constructed around each pixel of the image. LBP, because of its superior performance in texture-based applications, has been adopted in biomedical image processing to analyze the micro-structure of different body organs in X-ray, CT, and MRI images [11]. However, there are certain factors, such as difficult lighting conditions, noisy conditions, image rotation, etc., that limit the performance of LBP. In light of this, a lot of variants have been proposed in the literature for CBMIR. The simplest extension of LBP is local ternary pattern (LTP) [12]. Unlike LBP, in which the neighbors are coded as either 0 or 1, LTP is as a three-valued code where the neighbors are coded as 0, 1, or −1. LTP has shown superior performance in comparison to LBP under noisy, aging, and non-uniform lighting conditions. However, scenarios like threshold selection limit its performance. Murala and Wu have worked extensively in CBMIR and have proposed several variants of LBP: local co-occurrence ternary pattern (LTCoP) [13] (rotation-invariant but computationally expensive), local mesh pattern (LMeP) [14] (enhanced edge information with high computational complexity), local mesh peak valley edge pattern (LMePVP) [15] (a ternary pattern based on first-order derivatives, i.e., local mesh peak edge pattern (LMePEP) and second-order derivatives, i.e., local mesh valley edge pattern (LMeVEP)), and spherical symmetric three-dimensional LTP (SS-3D-LTP) [16] (primarily an extension of LTP from 2D to 3D). On similar grounds, Dubey et al. have proposed several LBP-based CBMIR variants such as local wavelet pattern (LWP) [17] (encodes the local inter-pixel relationship in the wavelet domain), local diagonal extrema pattern (LDEP) [18] (low-dimensional pattern incorporating only diagonal relationships among neighboring pixels), local bit-plane dissimilarity pattern (LBDISP) [19], and local bit-plane decoded pattern (LBDP) [20]) (decomposes an image into bit planes and forms a resultant feature vector by combining the local dissimilarity at each bit plane). Deep et al. also proposed two new CBMIR methods: directional local ternary quantized extrema pattern (DLTerQEP) (encodes relationship along three selected directions of mesh patterns) and local mesh ternary pattern (LMeTP) (encodes relationship along horizontal, vertical, diagonal, and anti-diagonal directional of local neighborhood) [21,22]. An improvement in retrieval performance of DLTerQEP has been presented as local quantized extrema quinary pattern (LQEQryP) [23]. Other promising variants of LBP used for retrieval and classification of facial images, texture images, etc., are local tetra pattern (LTP) [24], local gradient hexa pattern [25], local tri-diagonal pattern (LTDP) [26], local neighborhood difference pattern (LNDP) [27], local neighborhood intensity pattern (LNIP) [28], local directional gradient pattern (LDGP) [29], local directional relation pattern (LDRP) [30], local directional ZigZag pattern (LDZP) [31], local jet pattern (LJP) [32], local morphological pattern (LMP) [33], multichannel local ternary co-occurrence pattern (MCLTCoP) [34], and scale-pattern adaptive local binary pattern (SPALBP) [35]. Recently, deep learning has gained significant popularity in the research community owing to its ability to synthesize automated feature representations without manual intervention. Deep learning is data-driven and automatically generates features for a given set of training data, unlike handcrafted methods that rely on domain knowledge for feature construction. This has led to its wide spread use in MIR applications for medical image analysis [36,37,38,39,40]. Undoubtedly, the retrieval performance of such deep learning methods has been observed to be much superior to the other handcrafted feature extraction methods. However, the downside with such methods is their dependence on the data. The performance of such systems is impaired if the amount of data is too small to train them effectively (even in the case of transfer learning).

### 1.3. Identified Gap and Contributions

Despite significant advancements in CBMIR techniques, existing methods often struggle with the effective retrieval of medical images due to the complexity and variability inherent in medical data. These challenges include the difficulty in accurately capturing and representing the subtle texture patterns in medical images, which are essential for diagnostic purposes. Additionally, many current systems rely heavily solely on either local features or global features or simple descriptors, which may not be sufficient to differentiate between similar yet diagnostically distinct images. The gap that this study addresses lies in the inadequacy of existing CBMIR methods to effectively utilize texture features for the retrieval of medical images. Current methods are often vulnerable to noise and lack the ability to represent features across multiple scales, which is critical for accurate image analysis. The inability to handle noise effectively and capture multi-scale information results in reduced retrieval accuracy, particularly on complex medical datasets. This limitation hampers the potential of CBMIR systems to assist healthcare professionals in making accurate diagnosis.

In light of the strengths and shortcomings of the above-mentioned methods and the current need to develop effective and efficient CBMIR CAD systems, an attempt has been made in this paper to put forth a CBMIR system encompassing the novel idea of extracting noise-resistant texture features at multiple scales from neutrosophic transformed images of input medical image. Neutrosophic sets, with their ability to encode “indeterminacy” alongside “truth” and “falsity”, have garnered tremendous success in areas as diverse as decision making, information retrieval, and artificial intelligence, etc. [41,42]. Consequently, their use in image processing- and computer vision-related applications has been gaining a lot of attention, especially in areas like medical diagnosis, pattern recognition, etc. [43,44,45]. This paper aligns with the similar interest of utilizing neutrosophic information to develop a computer-assisted diagonsis system based on CBMIR. The key contributions of the paper are as follows:

1. A new idea of using neutrosophic information for extracting underlying textures from medical images. Neutrosophic images offer flexibility in representing texture information by allowing each pixel to have varying degrees of truth, indeterminacy, and falsity. This flexibility accommodates the diverse and complex nature of texture patterns in medical images, providing a more adaptable framework for feature extraction.
2. A new approach, MsNrRiTxP, is presented that extracts texture features from all three neutrosophic images, i.e., truth (*T*), indeterminacy (*I*), and falsity (*F*). The texture features from each of the *T*, *I*, and *F* images are appended together to form the final feature vector for MsNrRiTxP.
3. The presented work delineates an innovative approach which exhibits a significant enhancement over the existing CBMIR approaches by integrating a comprehensive set of features, i.e., noise resilience, rotation invariance, local and neighborhood information embedding, global information embedding, multi-scale feature representation, etc., under one umbrella. This approach is distinguished by its holistic one-stop solution strategy, which seamlessly amalgamates multiple traits into a singular, cohesive technique.
4. The proposed approach demonstrates superior retrieval performance by significantly outperforming the existing state-of-the-art LBP-based CBMIR and texture feature extraction approaches on four standard medical test datasets. To further substantiate the effectiveness of the proposed approach, an additional set of experiments is performed on noisy images of four test datasets.

The remainder of the paper is organised in the following manner: A detailed explanation of our proposed MsNrRiTxP texture descriptor is given in Section 2. The experimental framework employed to test the retrieval performance of the proposed and the compared techniques is presented in Section 3. The experimental findings on four test medical datasets are shown in Section 4. Lastly, Section 5 concludes the presented work.

## 2. Proposed Multi-Scale Noise-Resistant Rotation-Invariant Texture Pattern (MsNrRiTxP) Approach

This section presents the detailed working and layout of the proposed approach. The proposed approach is built from multiple sub-modules, which are detailed below.

1. Firstly, the medical image is transformed to the neutrosophic domain, such that for every input medical image, we obtain three neutrosophic images, i.e., truth (*T*), indeterminacy (*I*), and falsity (*F*).
2. Secondly, from each of the *T*, *I*, and *F* images, rotation-invariant and noise-robust texture feature pattern descriptors, $MsNrRiTxP_{r}^{T}$, $MsNrRiTxP_{r}^{I}$, and $MsNrRiTxP_{r}^{F}$ are extracted. The computation of the proposed pattern is based on construction of a symmetric neighborhood of 8r members around every pixel at a distance *r* from it. The parameter *r* also determines the spatial scale of the $MsNrRiTxP_{r}^{T}$, $MsNrRiTxP_{r}^{I}$, and $MsNrRiTxP_{r}^{F}$ patterns, which produces a constant dimensionality histogram at any spatial scale *r* with 8r sampling points for each neutrosophic image. In our work, texture features are extracted at multiples scales to capture the multi-resolution view of the image.
3. Lastly, the final $MsNrRiTxP_{r}^{\left\{T,I,F\right\}}$ pattern is formed by scale-wise appending of the individual patterns $MsNrRiTxP_{r}^{T}$, $MsNrRiTxP_{r}^{I}$, and $MsNrRiTxP_{r}^{F}$ extracted from the *T*, *I*, and *F* images, respectively. In other words, the $MsNrRiTxP_{r}^{\left\{T,I,F\right\}}$ pattern is formed by appending the patterns $MsNrRiTxP_{1}^{\left\{T,I,F\right\}}$, $MsNrRiTxP_{2}^{\left\{T,I,F\right\}}$, $MsNrRiTxP_{3}^{\left\{T,I,F\right\}}$, and so on, where each $MsNrRiTxP_{i}^{\left\{T,I,F\right\}}$ pattern is obtained by concatenating the patterns $MsNrRiTxP_{i}^{T}$, $MsNrRiTxP_{i}^{I}$, and $MsNrRiTxP_{i}^{F}$.

### 2.1. Construction of Neutrosophic Images

Neutrosophic sets, developed as a generalization of fuzzy sets, extend classical binary logic to embrace uncertainty and inconsistency [41]. They capture not just whether something belongs to a set (like “true”) or not (like “false”), but also the degree of indeterminacy or in betweenness. These sets incorporate three degrees of membership: truth (*T*), indeterminacy (*I*), and falsity (*F*). Each element belongs to these categories with independent values ranging from 0 to 1, allowing for more nuanced representations of data than traditional sets. A neutrosophic set NS is represented in the form, $NS=\langle \mu_{NS}\left(x\right),\sigma_{NS}\left(x\right),\tau_{NS}\left(x\right)\rangle$, where $\mu_{NS}\left(x\right)$, $\sigma_{NS}\left(x\right)$, and $\tau_{NS}\left(x\right)$ represent the degree of membership function, the degree of indeterminacy, and the degree of non-membership, respectively, for each element *x* (x∈X, where *X* is a non-empty fixed set) to the set NS.

**Figure 1:**
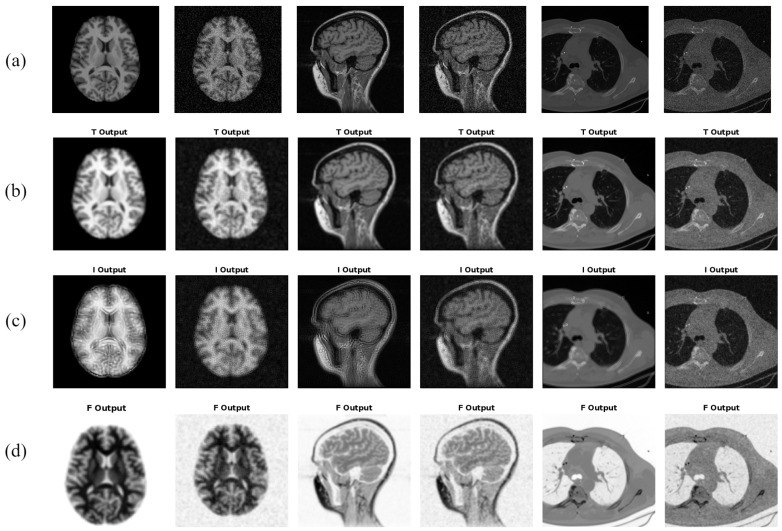
Neutrosophic images of input medical image when transformed into neutrosophic domain: (**a**) Sample noise-free and noisy medical images, (**b**) truth image ($T_{NS}$), (**c**) indeterminacy image ($I_{NS}$), and (**d**) falsity image ($F_{NS}$).

Building on this domain knowledge, a neutrosophic image $Z_{NS}$ is characterized by $T_{NS}$, $I_{NS}$, and $F_{NS}$ membership sets [43]. A pixel *P* (i.e., $P=Z_{NS}\left(i,j\right)$) in the neutrosophic domain can be represented as P=〈t,i,f〉, which reflects that the pixel is t% true, i% indeterminate, and f% false, where $t=T_{NS}\left(i,j\right)$, $i=I_{NS}\left(i,j\right)$, and $f=F_{NS}\left(i,j\right)$. Thus, a pixel Z(i,j) of an (original) image *Z* is transformed into the neutrosophic domain as $Z_{NS}\left(i,j\right)=\langle T_{NS}\left(i,j\right),I_{NS}\left(i,j\right),F_{NS}\left(i,j\right)\rangle$, where $T_{NS}\left(i,j\right)$, $I_{NS}\left(i,j\right)$, and $F_{NS}\left(i,j\right)$ are the membership values belonging to the membership sets; truth, indeterminacy, and falsity, respectively. The neutrosophic transformation of the original image *Z* into three neutrosophic domain images $T_{NS}$, $I_{NS}$, and $F_{NS}$ is particularly well suited for medical image analysis. Medical images, like X-rays or MRIs, often hold vital information encoded in their textures. These images can be inherently ambiguous, due to factors like noise, or subtle variations in tissue density. Improper handling of this uncertainty often leads to inaccurate diagnosis or missed interpretations. Neutrosophic sets, with their ability to encode indeterminacy alongside truth and falsity, can better capture these nuances, leading to more robust analysis. The mathematical notations to derive the three neutrosophic domain images $T_{NS}$, $I_{NS}$, and $F_{NS}$ are given below:

(1) $$T_{NS}\left(i,j\right)=\frac{\bar{Z}\left(i,j\right)-{\bar{Z}}_{\min}}{{\bar{Z}}_{\max}-{\bar{Z}}_{\min}}$$

(2) $$\bar{Z}\left(i,j\right)=\frac{1}{w\times w}\sum_{m=i-w/2}^{i+w/2}\sum_{n=j-w/2}^{j+w/2}Z\left(m,n\right)$$

(3) $$I_{NS}\left(i,j\right)=\frac{\delta \left(i,j\right)-\delta_{\min}}{\delta_{\max}-\delta_{\min}}$$

(4) $$\delta \left(i,j\right)=abs(Z\left(i,j\right)-\bar{Z}\left(i,j\right))$$

(5) $$F_{NS}\left(i,j\right)=1-T_{NS}\left(i,j\right)$$

where for every (i,j)th pixel, Z(i,j) represents its intensity value in the original image *Z*, $\bar{Z}\left(i,j\right)$ represents the mean intensity value in the w×w local neighborhood centered around it, and δ(i,j) represents the absolute of the difference between its intensity and its local mean value. Figure 1 shows the $T_{NS}$, $I_{NS}$, and $F_{NS}$ images obtained by the neutrosophic transformation of different samples of medical images.

### 2.2. Proposed MsNrRiTxP Pattern Descriptor

The fundamental design behind the working of the proposed approach has been drawn from the LBP operator [10]. Similar to LBP, the proposed approach captures the spatial structure of a local image texture in $T_{NS}$, $I_{NS}$, and $F_{NS}$ images by constructing a circular symmetric neighborhood centered around every pixel of the image. This allows the multi-resolution analysis of the image and enables the extraction of rotation-invariant features from them. Formally, given a pixel $p_{c}$ of the input image *P*, where $P\in \{T_{NS},I_{NS},F_{NS}\}$, a circular symmetric neighborhood is constructed around it at a distance *r*. Now, on this circular neighborhood, corresponding to distance parameter *r*, 8r neighboring pixels of $p_{c}$ are sampled that are evenly distributed along this circle of radius *r*. Assuming the center pixel, i.e., $p_{c}$, to be at origin (0,0), the coordinates of the neighboring pixels are given by (−rsin(2πn)/8r,rcos(2πn)/8r). The gray values of neighboring pixels which do not fall exactly at the center of pixels are estimated by interpolation. For instance, a total of 8, 16, 24, etc., neighboring pixels of $p_{c}$ will be sampled for circular neighborhoods at distances r=1,2,3, respectively, from $p_{c}$. Let $p_{r}^{\left(i,j\right)}$ represent the neighbor vector of pixel $p_{c}$ (located at the (i,j)th location in image *P*), mentioning its 8r neighboring pixels.

(6) $$p_{r}^{\left(i,j\right)}=[p_{\left(r,0\right)}^{\left(i,j\right)},\ldots ,p_{\left(r,8r-1\right)}^{\left(i,j\right)}]$$

In our work, three different forms of binary patterns, i.e., MsTrP, NrTxP, and RiTxP are computed for every pixel of three neutrosophic images $T_{NS}$, $I_{NS}$, and $F_{NS}$ using their neighbor vectors $p_{r}^{\left(i,j\right)}$. The detailed process of computing these patterns is described in the following subsections.

#### 2.2.1. Pattern 1: MsTrP

In the computation of this pattern, the neighbor vector $p_{r}^{\left(i,j\right)}$ containing 8r elements corresponding to a circular neighborhood at distance *r* from $p_{c}$ is transformed to the median quantized neighbor vector ${\mathrm{mqp}}_{r}^{\left(i,j\right)}$ by applying the median filter along the arc to restrict its count of elements to 8. In other words, irrespective of the scale of the input image (determined by the value of the *r* parameter), the median quantized neighbor vector ${\mathrm{mqp}}_{r}^{\left(i,j\right)}$ always consists of 8 elements. The following table (Table 1) illustrates this fact with suitable examples.

The median quantized neighbor vector ${\mathrm{mqp}}_{r}^{\left(i,j\right)}$ is defined as

(7) $${\mathrm{mqp}}_{r}^{\left(i,j\right)}=[mqp_{\left(r,0\right)}^{\left(i,j\right)},\ldots ,mqp_{\left(r,7\right)}^{\left(i,j\right)}]$$

where

(8) $$\begin{array}{cc} mqp_{\left(r,k\right)}^{\left(i,j\right)}=MEDIAN([p_{\left(r,rk\right)}^{\left(i,j\right)},\ldots ,p_{\left(r,rk+t\right)}^{\left(i,j\right)}]), & \\ & k\in \{0,1,\ldots ,7\},\;t\in \{0,\ldots ,r-1\} \end{array}$$

**Figure 2:**
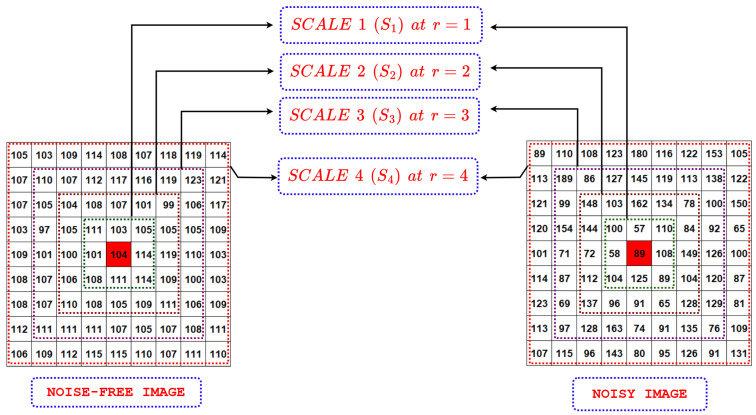
A sample image patch (around a center pixel $p_{c}$, highlighted in red) from noise-free and noisy image for illustration of noise robustness of the ${TrP}_{r}$ pattern. The figure also shows the multi-resolution view of the image patches at four scales $S_{1}$, $S_{2}$, $S_{3}$, and $S_{4}$, corresponding to r=1,2,3,4, respectively.

**Figure 3:**
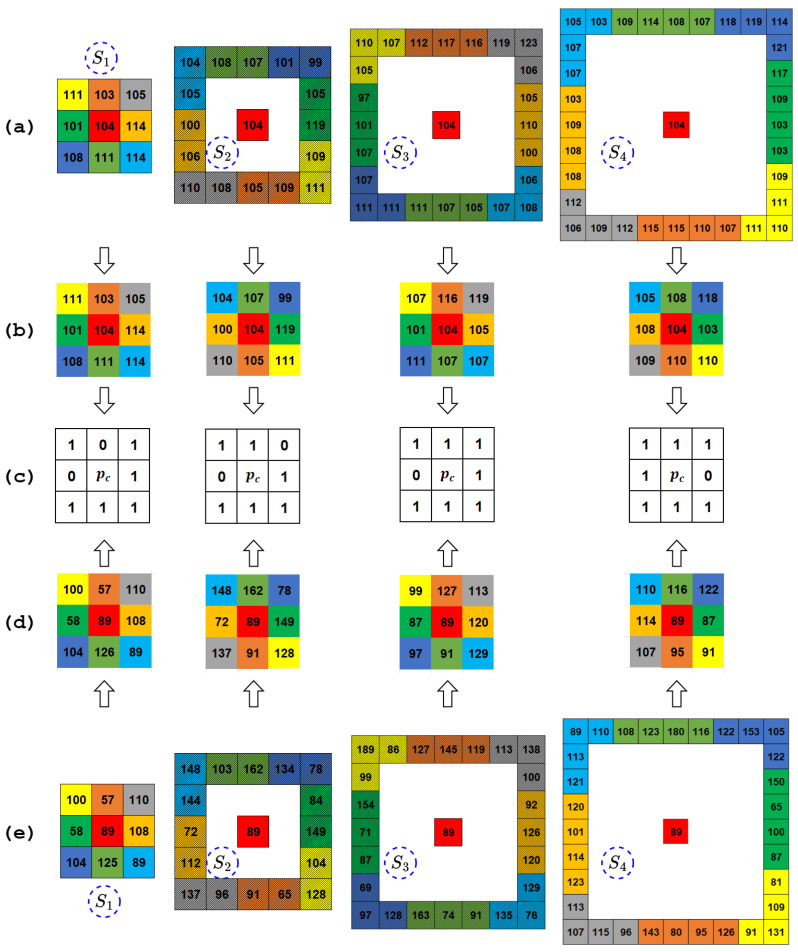
Example illustrating the computation of proposed ${TrP}_{r}$ pattern for center pixel $p_{c}$ (highlighted in RED color) at multiples scales $S_{1}$, $S_{2}$, $S_{3}$, and $S_{4}$, corresponding to r=1,2,3,4, respectively, on noise-free and noisy image patch shown in Figure 2: (**a**) Neighbor vectors $p_{r}$ at scales $S_{1}$, $S_{2}$, $S_{3}$, and $S_{4}$ for noise-free image patch; (**b**) median quantized neighbor vectors ${\mathrm{mqp}}_{r}$ at scales $S_{1}$, $S_{2}$, $S_{3}$, and $S_{4}$ for noise-free image patch; (**c**) proposed ${TrP}_{r}$ binary pattern; (**d**) median quantized neighbor vectors ${\mathrm{mqp}}_{r}$ at scales $S_{1}$, $S_{2}$, $S_{3}$, and $S_{4}$ for noisy image patch; (**e**) neighbor vectors $p_{r}$ at scales $S_{1}$, $S_{2}$, $S_{3}$, and $S_{4}$ for noisy image patch.

Thus, given ${\mathrm{mqp}}_{r}^{\left(i,j\right)}$, a local binary pattern descriptor with respect to the center pixel $p_{c}$ is computed as follows:

(9) $${TrP}_{r}^{\left(i,j\right)}=\sum_{n=0}^{7}s\left(mqp_{\left(r,n\right)}^{\left(i,j\right)}-p_{c}\right)2^{n},\;s\left(x\right)=\left\{\begin{array}{cc} 1 & x\geq 0\\ 0 & x<0 \end{array}\right.$$

where s() is the sign function. It can be easily observed that for any parameter *r* there will always be $2^{8}=256$${TrP}_{r}$ patterns in total. Furthermore, the transformation of neighbor vectors from $p_{r}$ to ${\mathrm{mqp}}_{r}$ makes the pattern more robust to noise, as illustrated in Figure 2 and Figure 3 with the help of a suitable example. Following the inspiration of rotation-invariant LBP in [46], the ${TrP}_{r}$ patterns are transformed to make them rotation-invariant and reduce the count of possible patterns (thereby, reducing the dimensionality) at any scale (i.e., for any value of *r* parameter) from 256 to 10. The transformed ${MsTrP}_{r}$ patterns are defined as follows:

(10) $${MsTrP}_{r}^{\left(i,j\right)}=\left\{\begin{array}{cc} \sum_{n=0}^{7}s\left(mqp_{\left(r,n\right)}^{\left(i,j\right)}-p_{c}\right) & \;\mathrm{if}\;U\left({TrP}_{r}^{\left(i,j\right)}\right)\leq 2\\ 9 & \;\mathrm{otherwise}\; \end{array}\right.$$

where the function U() reflects the use of rotation-invariant uniform patterns having at most two transitions in bit value (i.e., from 1 to 0 or from 0 to 1) along the neighbors. Thus, exactly 9 uniform binary patterns exists which will be assigned labels from {0,1,…,7,8} corresponding to a cardinality of 1 in the bit pattern. All remaining non-uniform bit patterns are assigned the label {9}.

(11) $$\begin{array}{cc} U\left({TrP}_{r}^{\left(i,j\right)}\right)=|s\left(mqp_{\left(r,7\right)}^{\left(i,j\right)}-p_{c}\right)- & s\left(mqp_{\left(r,0\right)}^{\left(i,j\right)}-p_{c}\right)|+\\ & \;\sum_{n=1}^{7}\left|s\left(mqp_{\left(r,n\right)}^{\left(i,j\right)}-p_{c}\right)-s\left(mqp_{\left(r,n-1\right)}^{\left(i,j\right)}-p_{c}\right)\right| \end{array}$$

Therefore, for three neutrosophic images $T_{NS}$, $I_{NS}$, and $F_{NS}$, assuming a size M×N, the ${MsTrP}_{r}$ pattern is computed for every pixel {(i,j)|i∈{1+r,…,M−r},j∈{1+r,…,N−r}}. Thus, $T_{NS}$, $I_{NS}$, and $F_{NS}$ are represented by the probability distribution (histogram) of the ${MsTrP}_{r}$ patterns as follows:

(12) $${MsTrP}_{r}^{T}\left(\eta \right)=\sum_{i=1+r}^{M-r}\sum_{j=1+r}^{N-r}\zeta \left(({MsTrP}_{r}^{\left(i,j\right)}{)}^{T},\eta \right),\;\eta \in \left\{0,1,\ldots ,8,9\right\}$$

(13) $${MsTrP}_{r}^{I}\left(\eta \right)=\sum_{i=1+r}^{M-r}\sum_{j=1+r}^{N-r}\zeta \left(({MsTrP}_{r}^{\left(i,j\right)}{)}^{I},\eta \right),\;\eta \in \left\{0,1,\ldots ,8,9\right\}$$

(14) $${MsTrP}_{r}^{F}\left(\eta \right)=\sum_{i=1+r}^{M-r}\sum_{j=1+r}^{N-r}\zeta \left(({MsTrP}_{r}^{\left(i,j\right)}{)}^{F},\eta \right),\;\eta \in \left\{0,1,\ldots ,8,9\right\}$$

where ζ is calculated by the following rule:

(15) $$\zeta \left(\alpha_{1},\alpha_{2}\right)=\left\{\begin{array}{cc} 1, & \;\mathrm{if}\;\alpha_{1}=\alpha_{2}\\ 0, & \;\mathrm{otherwise}.\; \end{array}\right.$$

#### 2.2.2. Pattern 2: NrTxP

This pattern quantizes the neighbor vector $p_{r}^{\left(i,j\right)}$ with respect to the magnitude of local differences in gray values of the neighboring pixels with the center pixel $p_{c}$, unlike the MsTrP pattern, where the quantization is performed with respect to the output of the median filter. In the NrTxP pattern, the neighbor vector $p_{r}^{\left(i,j\right)}$ is initially transformed to the local differences neighbor vector ${\mathrm{ldp}}_{r}^{\left(i,j\right)}$ by taking the absolute value of the local differences between the center pixel $p_{c}$ and its neighboring pixels, as shown below:

(16) $${\mathrm{ldp}}_{r}^{\left(i,j\right)}=[{ldp}_{\left(r,0\right)}^{\left(i,j\right)},\ldots ,{ldp}_{\left(r,8r-1\right)}^{\left(i,j\right)}]$$

where

(17) $${ldp}_{r,k}^{\left(i,j\right)}=|p_{r,k}^{\left(i,j\right)}-p_{c}|,\;k\in \left\{0,1,\ldots ,8r-1\right\}$$

Now, the local differences neighbor vector ${\mathrm{ldp}}_{r}^{\left(i,j\right)}$ is quantized to obtain the mean local differences quantized neighbor vector ${\mathrm{mldqp}}_{r}^{\left(i,j\right)}$ by averaging the absolute value of the local differences along the arc to restrict its cardinality to 8. Similar to ${\mathrm{mqp}}_{r}^{\left(i,j\right)}$, the count of elements in the ${\mathrm{mldqp}}_{r}^{\left(i,j\right)}$ neighbor vector will always be 8, irrespective of the scale of the neighborhood (or the value of parameter *r*). The idea behind averaging the local differences is to induce noise robustness capability in the NrTxP pattern. By averaging the local difference, the impact of noise in the local neighborhood is significantly reduced.

The mean local differences quantized neighbor vector ${\mathrm{mldqp}}_{r}^{\left(i,j\right)}$ is defined as

(18) $${\mathrm{mldqp}}_{r}^{\left(i,j\right)}=[{mldqp}_{\left(r,0\right)}^{\left(i,j\right)},\ldots ,{mldqp}_{\left(r,7\right)}^{\left(i,j\right)}]$$

where

(19) $${mldqp}_{\left(r,k\right)}^{\left(i,j\right)}=\frac{1}{r}\sum_{t=0}^{r-1}{ldp}_{r,rk+t}^{\left(i,j\right)},\;k\in \left\{0,1,\ldots ,7\right\}$$

Similar to ${TrP}_{r}$, the second local binary pattern descriptor ${TxP}_{r}$, with respect to the center pixel $p_{c}$, is computed using the mean local differences quantized neighbor vector, as shown below:

(20) $${TxP}_{r}^{\left(i,j\right)}=\sum_{n=0}^{7}s\left({mldqp}_{\left(r,n\right)}^{\left(i,j\right)}-\nu_{r}\right)2^{n},\;s\left(x\right)=\left\{\begin{array}{cc} 1 & x\geq 0\\ 0 & x<0 \end{array}\right.$$

where $\nu_{r}$ is defined as

(21) $$\nu_{r}=\sum_{y=j-r}^{j+r}{\mu ld}_{r}^{\left((i-r),y\right)}+\sum_{y=j-r}^{j+r}{\mu ld}_{r}^{\left((i+r),y\right)}\;+\sum_{x=i-r+1}^{i+r-1}{\mu ld}_{r}^{\left(x,(j-r)\right)}+\sum_{x=i-r+1}^{i+r-1}{\mu ld}_{r}^{\left(x,(j+r)\right)}$$

for i∈{1+r,…,M−r}, j∈{1+r,…,N−r}. Also, ${\mu ld}_{r}$ is the mean local differences image, obtained as follows:

(22) $${\mu ld}_{r}^{\left(i,j\right)}=\frac{1}{\left(8r\right)}\sum_{n=0}^{8r-1}{ldp}_{\left(r,n\right)}^{\left(i,j\right)},\;i\in \left\{1+r,\ldots ,M-r\right\}\;j\in \left\{1+r,\ldots ,N-r\right\}$$

The ${TxP}_{r}$ patterns are then made noise-resistant using the same transformation as adopted in the case of the ${TrP}_{r}$ patterns. Thus, like the ${MsTrP}_{r}$ patterns, the dimensionality of the transformed ${TxP}_{r}$ patterns, i.e., ${NrTxP}_{r}$, is always 10, irrespective of the scale of the neighborhood (or the value of parameter *r*). The ${NrTxP}_{r}$ patterns are defined as follows:

(23) $${NrTxP}_{r}^{\left(i,j\right)}=\left\{\begin{array}{cc} \sum_{n=0}^{7}s\left({mldqp}_{\left(r,n\right)}^{\left(i,j\right)}-\nu_{r}\right) & \;\mathrm{if}\;U\left({TxP}_{r}^{\left(i,j\right)}\right)\leq 2\\ 9 & \;\mathrm{otherwise}\; \end{array}\right.$$

Summarizing, for the neutrosophic images $T_{NS}$, $I_{NS}$, and $F_{NS}$, the probability distribution (histogram) of the ${NrTxP}_{r}$ patterns for the three images at scale *r* are given as

(24) $${NrTxP}_{r}^{T}\left(\eta \right)=\sum_{i=1+r}^{M-r}\sum_{j=1+r}^{N-r}\zeta \left(({NrTxP}_{r}^{\left(i,j\right)}{)}^{T},\eta \right),\;\eta \in \left\{0,1,\ldots ,8,9\right\}$$

(25) $${NrTxP}_{r}^{I}\left(\eta \right)=\sum_{i=1+r}^{M-r}\sum_{j=1+r}^{N-r}\zeta \left(({NrTxP}_{r}^{\left(i,j\right)}{)}^{I},\eta \right),\;\eta \in \left\{0,1,\ldots ,8,9\right\}$$

(26) $${NrTxP}_{r}^{F}\left(\eta \right)=\sum_{i=1+r}^{M-r}\sum_{j=1+r}^{N-r}\zeta \left(({NrTxP}_{r}^{\left(i,j\right)}{)}^{F},\eta \right),\;\eta \in \left\{0,1,\ldots ,8,9\right\}$$

#### 2.2.3. Pattern 3: RiTxP

Lastly, to construct this pattern, the center pixel $p_{c}$ is encoded into one of the two bins formed by thresholding its gray value against the local mean gray value ($\mu_{r}$) in the neighborhood of $p_{c}$ at scale *r*. Thus, the dimensionality of the histogram formed from the RiTxP patterns will always be 2, irrespective of the scale of the neighborhood (or the value of parameter *r*).

(27) $${RiTxP}_{r}^{\left(i,j\right)}=s\left(p_{c}-\mu_{r}\right),\;s\left(x\right)=\left\{\begin{array}{cc} 1 & x\geq 0\\ 0 & x<0 \end{array}\right.$$

where $\mu_{r}$ is defined as

(28) $$\mu_{r}=\sum_{y=j-r}^{j+r}p_{r}^{\left((i-r),y\right)}+\sum_{y=j-r}^{j+r}p_{r}^{\left((i+r),y\right)}\;+\sum_{x=i-r+1}^{i+r-1}p_{r}^{\left(x,(j-r)\right)}+\sum_{x=i-r+1}^{i+r-1}p_{r}^{\left(x,(j+r)\right)}$$

For neutrosophic images $T_{NS}$, $I_{NS}$, and $F_{NS}$, the probability distribution (histogram) of the ${RiTxP}_{r}$ patterns at scale *r* is given as

(29) $${RiTxP}_{r}^{T}\left(\eta \right)=\sum_{i=1+r}^{M-r}\sum_{j=1+r}^{N-r}\zeta \left(({RiTxP}_{r}^{\left(i,j\right)}{)}^{T},\eta \right),\;\eta \in \left\{0,1\right\}$$

(30) $${RiTxP}_{r}^{I}\left(\eta \right)=\sum_{i=1+r}^{M-r}\sum_{j=1+r}^{N-r}\zeta \left(({RiTxP}_{r}^{\left(i,j\right)}{)}^{I},\eta \right),\;\eta \in \left\{0,1\right\}$$

(31) $${RiTxP}_{r}^{F}\left(\eta \right)=\sum_{i=1+r}^{M-r}\sum_{j=1+r}^{N-r}\zeta \left(({RiTxP}_{r}^{\left(i,j\right)}{)}^{F},\eta \right),\;\eta \in \left\{0,1\right\}$$

#### 2.2.4. Final Construction of MsNrRiTxP Pattern Descriptor

The final histogram of the proposed MsNrRiTxP pattern is constructed from the joint histograms of the three pattern descriptors, i.e., MsTrP, NrTxP, and RiTxP. The joint histogram of MsTrP, NrTxP, and RiTxP, i.e., MsTrP×NrTxP×RiTxP has a very high dimensionality of 200 features (10×10×2) at every scale. Accordingly, if five scales have been considered to perform multi-resolution analysis of the input image, the total number of features will accumulate to 1000(5×200), which is very high considering we have three neutrosophic images (corresponding to every input image) to work with. Therefore, in our work, instead of taking the joint histogram of all three patterns, the joint histogram of MsTrP×RiTxP is concatenated with the joint histogram of NrTxP×RiTxP, thereby reducing the dimensionality of the MsNrRiTxP pattern to 40(10×2+10×2) bins at every scale. The sequence of concatenation adopted for the construction of the MsNrRiTxP pattern for the input (medical) image at a single scale (i.e., for a particular value of *r*) is explained below.

(32) $${MsNrRiTxP}_{r}^{\left\{T,I,F\right\}}={MsNrRiTxP}_{r}^{T}\;\|\;{MsNrRiTxP}_{r}^{I}\;\|\;{MsNrRiTxP}_{r}^{F}$$

where ‖⇒ConcatenationOperator and

(33) $${MsNrRiTxP}_{r}^{T}={MsRiTxP}_{r}^{T}\;\|\;{NrRiTxP}_{r}^{T},$$

(34) $${MsNrRiTxP}_{r}^{I}={MsRiTxP}_{r}^{I}\;\|\;{NrRiTxP}_{r}^{I},$$

(35) $${MsNrRiTxP}_{r}^{F}={MsRiTxP}_{r}^{F}\;\|\;{NrRiTxP}_{r}^{F},$$

where MsRiTxP and NrRiTxP are joint histograms defined as

(36) $$\begin{array}{c} {MsRiTxP}_{r}^{T}={MsTrP}_{r}^{T}\;⊗\;{RiTxP}_{r}^{T},\\ {NrRiTxP}_{r}^{T}={NrTxP}_{r}^{T}\;⊗\;{RiTxP}_{r}^{T}, \end{array}$$

(37) $$\begin{array}{c} {MsRiTxP}_{r}^{I}={MsTrP}_{r}^{I}\;⊗\;{RiTxP}_{r}^{I},\\ {NrRiTxP}_{r}^{I}={NrTxP}_{r}^{I}\;⊗\;{RiTxP}_{r}^{I}, \end{array}$$

(38) $$\begin{array}{c} {MsRiTxP}_{r}^{F}={MsTrP}_{r}^{F}\;⊗\;{RiTxP}_{r}^{F},\\ {NrRiTxP}_{r}^{F}={NrTxP}_{r}^{F}\;⊗\;{RiTxP}_{r}^{F}, \end{array}$$

The MsNrRiTxP pattern for multi-scale resolutions, i.e., for different values of *r* (r=1,2,3,…), is constructed by concatenating the single-scale ${MsNrRiTxP}_{r}$ patterns, as described below:

(39) $$\begin{array}{cc} {MsNrRiTxP}_{\left\{r=1,2,\ldots ,S\right\}}^{\left\{T,I,F\right\}}= & {MsNrRiTxP}_{1}^{\left\{T,I,F\right\}}\;\|\;\\ & \;\;{MsNrRiTxP}_{2}^{\left\{T,I,F\right\}}\;\ldots \;\|\;\ldots \;\|\;{MsNrRiTxP}_{S}^{\left\{T,I,F\right\}} \end{array}$$

## 3. Experimental Setup

This section outlines the computational framework adopted to evaluate the effectiveness of our proposed methodology in contrast to recent and state-of-the-art retrieval techniques detailed in Table 2. To ensure fairness, all the methods were implemented in MATLAB 2020b.

### 3.1. Similarity Measure

The effectiveness of any CBMIR system relies extensively on the selection of a strong similarity measure to compare the feature vector of the query image with the feature vectors of database images. The extended Canberra distance [47] is a commonly used and popular similarity measure in retrieval applications. The mathematical expression for the extended Canberra distance is given by

(40) $$D_{ECD}\left(t,Q\right)=\sum_{\tau =1}^{\dim}\frac{\left|F^{Q}\left(\tau \right)-F^{t}\left(\tau \right)\right|}{\left(F^{Q}\left(\tau \right)+\mu^{Q}\right)+\left(F^{t}\left(\tau \right)+\mu^{t}\right)}$$

where

(41) $$\mu^{Q}=\frac{1}{\dim}\sum_{\tau =1}^{\dim}F^{Q}\left(\tau \right),\;\mu^{t}=\frac{1}{\dim}\sum_{\tau =1}^{\dim}F^{t}\left(\tau \right)$$

and $F^{Q}$ and $F^{t}$ are the feature vectors of query image *Q* and database image *t*, respectively.

### 3.2. Performance Measures

All of the images in the database were used as query images in our experiments. The following four performance metrics, i.e., average precision rate (avgP), average retrieval rate (avgR), F-score $\left(F_{score}\right)$, and mean average precision (MavgP) have been employed to evaluate the effectiveness of every retrieval method.

$$avgP\left(\%\right)=\frac{\;\mathrm{number}\;\mathrm{of}\;\mathrm{relevant}\;\mathrm{images}\;\mathrm{retrieved}\;}{\;\mathrm{total}\;\mathrm{number}\;\mathrm{of}\;\mathrm{images}\;\mathrm{retrieved}\;(\eta )}$$

$$avgR\left(\%\right)=\frac{\;\mathrm{number}\;\mathrm{of}\;\mathrm{relevant}\;\mathrm{images}\;\mathrm{retrieved}\;}{\;\mathrm{total}\;\mathrm{number}\;\mathrm{of}\;\mathrm{relevant}\;\mathrm{images}\;\mathrm{in}\;\mathrm{the}\;\mathrm{database}\;}$$

(42) $$avgP\left(\%\right)=\frac{100}{\omega }\sum_{i=1}^{\omega }\frac{r\left(DB_{i}\right)}{\eta }$$

(43) $$avgR\left(\%\right)=\frac{100}{\omega }\sum_{i=1}^{\omega }\frac{r\left(DB_{i}\right)}{g\left(DB_{i}\right)}$$

(44) $$F_{score}\left(\%\right)=\frac{2\times avgP\times avgR}{avgP+avgR}$$

(45) $$MavgP\left(\%\right)=\frac{100}{\omega }\sum_{i=1}^{\omega }\sum_{\eta =1}^{g\left(DB_{i}\right)}\frac{r\left(DB_{i}\right)}{\eta }$$

where ω denotes the image count in the database DB, and $r\left(DB_{i}\right)$ and $r\left(DB_{i}\right)$ are the number of relevant images retrieved and the number of relevant ground truth images available for the *i*th query image, respectively.

### 3.3. Dataset Description

**Figure 4:**
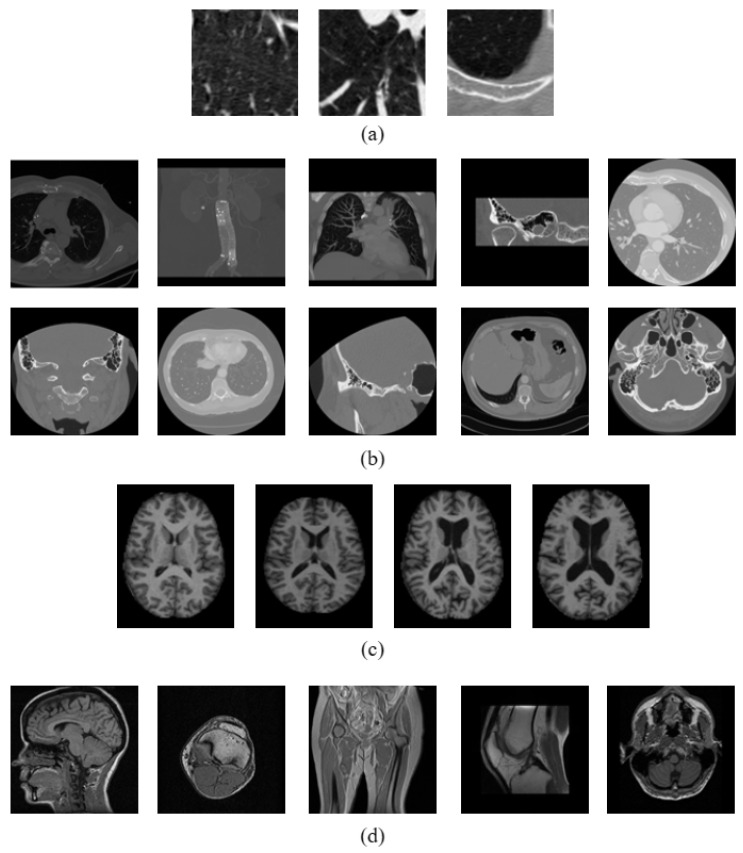
Sample image from each class of (**a**) Emphysema CT database, (**b**) NEMA CT database, (**c**) OASIS MRI database, and (**d**) NEMA MRI database.

Four test datasets were considered in our experimental framework to test the retrieval prowess of the proposed and the compared approaches. These include the Emphysema CT database [48] and the NEMA CT database for the purpose of retrieving CT images. Additionally, the OASIS MRI database [49] and the NEMA MRI database were used for the retrieval of MRI images. A summary of these datasets is given in Table 3. The intent of these datasets is to evaluate the effectiveness of the proposed and compared approaches to encode a multitude of textural information present in CT and MR images. Additionally, the experiments assess the methods’ effectiveness in representing changes in shape at both global and local scales. The Emphysema CT database and OASIS MRI database both consist of images depicting specific anatomical regions, namely, the lung and brain, respectively. Therefore, in order to achieve a high level of retrieval performance on these datasets, it is necessary for a method to possess the capability to effectively differentiate between images that may appear identical in general but actually differ significantly due to the local variations in their shapes. However, when it comes to the NEMA CT and NEMA MRI databases, which consist of images of various body parts, the method must possess superior information representation capability to effectively distinguish between images that have very distinct overall representations, particularly on a global scale. A sample image from each class of the four test datasets is shown in Figure 4.

**Figure 5:**
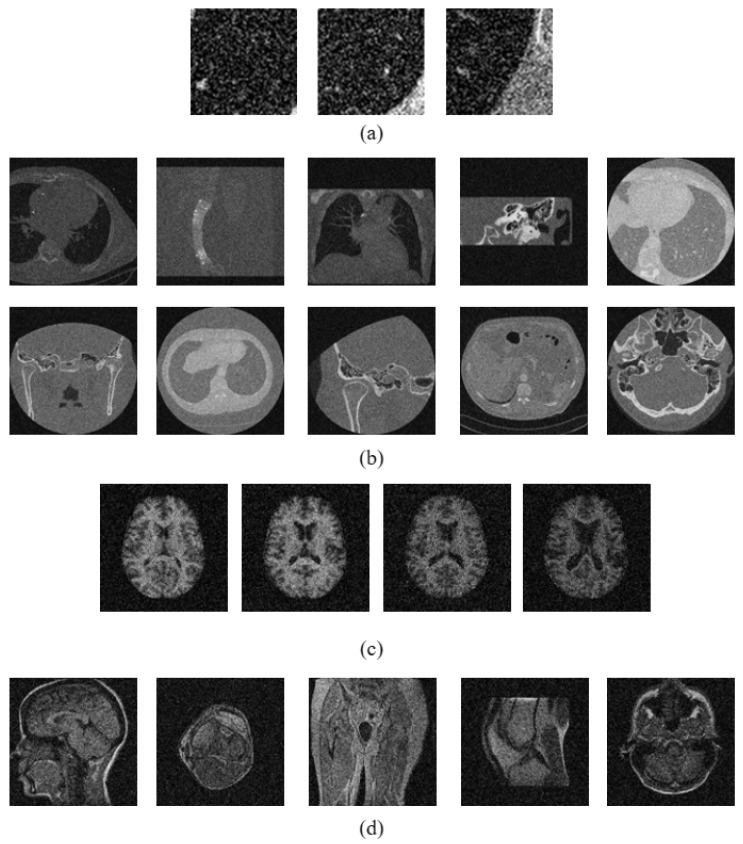
Sample noisy image from each class of (**a**) Emphysema CT database, (**b**) NEMA CT database, (**c**) OASIS MRI database, and (**d**) NEMA MRI database.

To further substantiate the superiority of the proposed approach over the existing methods, an additional set of experiments performed under noisy conditions on these datasets were performed in this paper. The noisy images were generated by introducing zero-mean additive white Gaussian noise with standard deviation varying between [5,50]. For the purpose of evaluating the noise robustness capability of the proposed and the compared approaches, the noise-free images were used as database images in these experiments, while the noisy images were used as query images. Figure 5 shows a sample noisy image from each class of the four test datasets.

## 4. Experimental Results and Discussions

The proposed method was tested on four test databases with both noise-free and noisy images and a comparison with the existing texture classification methods mentioned in Table 2 is presented in this section. The proposed MsNrRiTxP pattern is computed at nine scales (i.e., r={1,2,…,9}) for the NEMA CT, NEMA MRI, and OASIS MRI databases. However, for the Emphysema CT database, the number of scales considered in our approach is five (i.e., r={1,2,…,5}). This is because the number of pixels in the Emphysema CT database images is much less (i.e., 61×61). For the sake of fairness, the parameter settings for the compared methods were kept as mentioned in their respective sources. For comparative analysis, the implementations of the compared methods available in the public domain were used wherever possible. In the case that the implementations were not available, we used our implementation of that method, developed as per our best understanding. For every query image, the retrieval results for the top 100 matches are tabulated here.

### 4.1. Performance Analysis on Noise-Free Images

Table 4, Table 5, Table 6 and Table 7 compares the retrieval rates on noise-free images of the four test datasets yielded by the proposed MsNrRiTxP and the compared approaches. From the tables, it is very evident that the proposed approach substantially surpassed all the compared approaches on all four test datasets using all four performance metrics. In the case of the Emphysema CT database, the proposed approach amassed average gains in retrieval rates of 12.61%, 7.25%, 9.21%, and 9.59% for avgR, avgP, $F_{score}$, and MavgP, respectively, over all the compared approaches. Among the compared approaches, LDGP demonstrated the worst retrieval performance of 63.64%, 35.55%, 45.61%, and 47.57%, lagging behind the proposed approach with substantial differences of 18.72%, 10.79%, 13.70%, and 16.25% in terms of avgR, avgP, $F_{score}$, and MavgP, respectively. On the other end, the recently proposed SPALBP approach showed excellent retrieval capability among the compared approaches, lagging behind our proposed MsNrRiTxP approach by approximately 1.00% in terms of all four performance metrics. In contrast to the Emphysema CT database that comprises CT images specifically focused on lung tissues, the NEMA CT database consists of CT images of different body parts. Due to the greater ease of distinguishing between CT images of various body parts compared to different lung tissues, the retrieval rates on the NEMA CT database were significantly higher than those on the Emphysema CT database. Table 5 clearly demonstrates that the proposed MsNrRiTxP approach surpassed all other methods, with an average increases in retrieval rates of 5.17%, 6.50%, 6.29%, and 2.83% for avgR, avgP, $F_{score}$, and MavgP, respectively. LBDISP demonstrated the worst retrieval performance, lagging behind the proposed approach by 27.57% (avgR), 23.91% (avgP), 26.02% ($F_{score}$), and 17.15% (MavgP). The multi-scale encoding of texture features enables our proposed approach to capture the underlining shape of the body organ distinctively, and therefore, it is able to effectively distinguish between shapes of different organs with pin-point accuracy.

**Figure 6:**
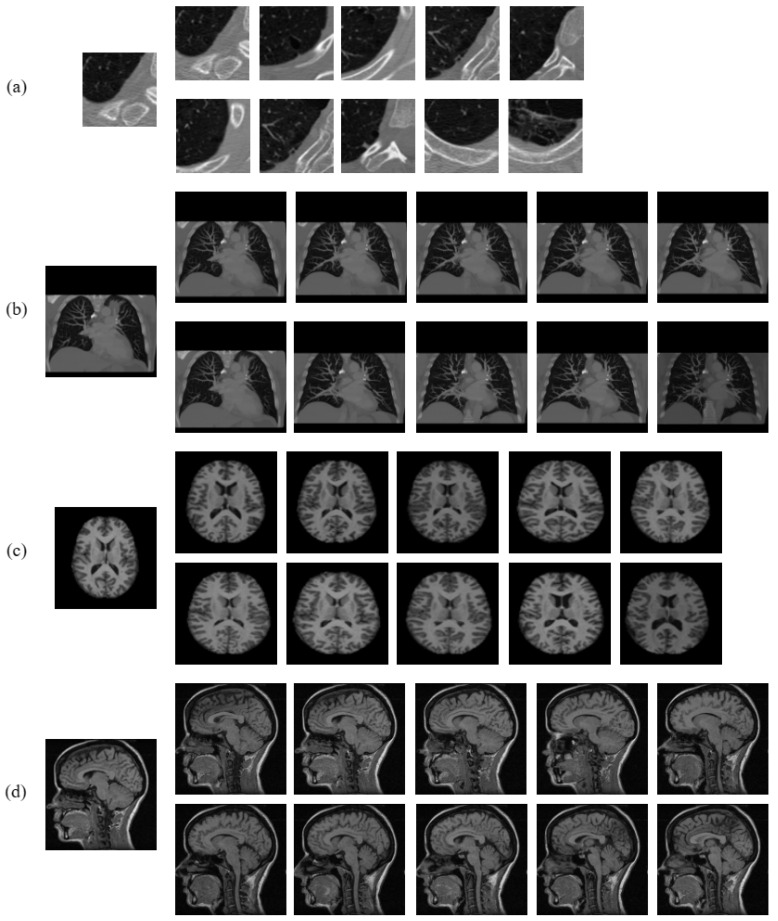
Query results of the proposed method for noise-free query images on (**a**) Emphysema CT database, (**b**) NEMA CT database, (**c**) OASIS MRI database, and (**d**) NEMA MRI database.

For the OASIS MRI dataset, the task of retrieving matching images similar in structure to the query image is more intricate compared to the NEMA MRI dataset. The images in the OASIS dataset pose a significant challenge due to their subtle inter-class variations. While the images may appear to be similar on the surface, they can be differentiated very minutely based on the shape of the ventricular area of the brain. In light of this, the retrieval rates attained on the OASIS dataset were lower compared to all of the other datasets. The retrieval performance was 42.93% (avgR), 44.52% (avgP), 43.71% ($F_{score}$), and 53.11% (MavgP) for the proposed MsNrRiTxP approach on the OASIS dataset, in comparison to 100% (avgR), 83.47% (avgP), 90.99% ($F_{score}$), and 100% (MavgP) attained on the NEMA MRI dataset. The proposed approach demonstrated clear superiority over all the compared approaches, thereby achieving remarkable improvements of 11.73% and 2.08% in avgR, 11.37% and 7.57% in avgP, 11.56% and 5.49% in $F_{score}$, and 14.60% and 0.83% in MavgP on the OASIS and NEMA MRI datasets, respectively. Despite the inherent difficulty in distinguishing the images of the OASIS dataset, the proposed approach showcased substantial gains with respect to the NEMA MRI dataset. This highlights the remarkable discriminatory power that the proposed approach possesses by encoding the complex intricacies present in the texture and shape data. With retrieval rates 28.49% (avgR), 30.43% (avgP), 30.08% ($F_{score}$), and 13.32% (MavgR) lower with respect to the proposed approach, LWP yielded the lowest retrieval rates among all the compared methods. The NEMA MRI database represents merely five classes, each class being associated with a different anatomical part of the body. Thus, there is a very little room for ambiguity when it comes to classifications, as the classes are clearly separated from each other. This leads to enhanced retrieval rates on the NEMA dataset in comparison to OASIS. The query results on all the test datasets are illustrated in Figure 6.

### 4.2. Performance Analysis on Noisy Images

**Figure 7:**
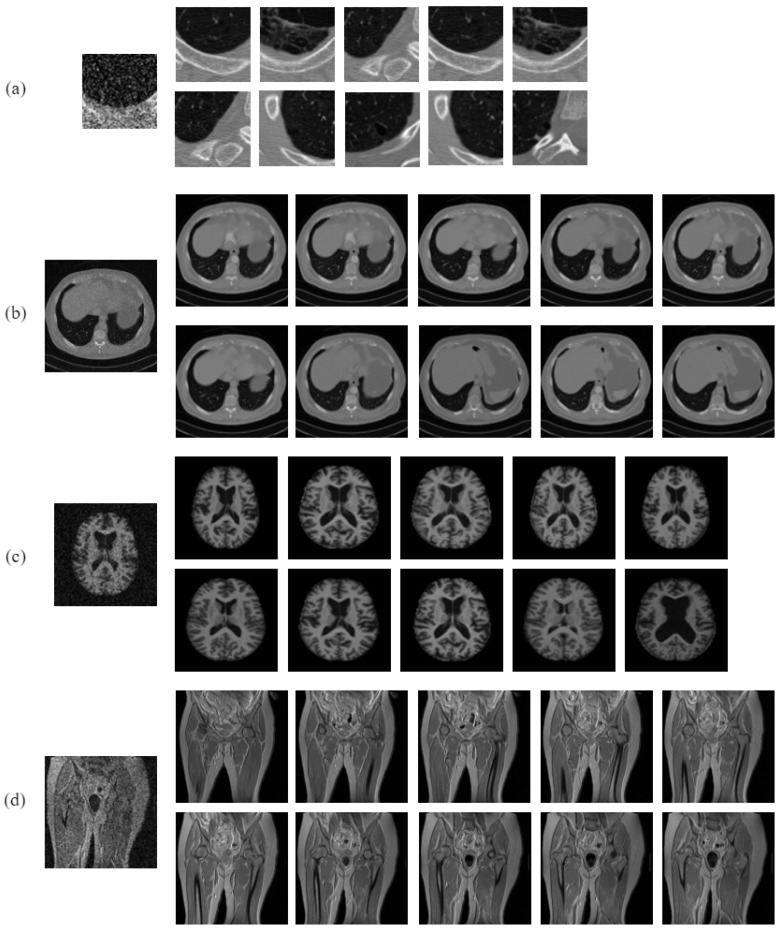
Query results of the proposed method for noisy query image on (**a**) Emphysema CT database, (**b**) NEMA CT database, (**c**) OASIS MRI database, and (**d**) NEMA MRI database.

**Figure 8:**
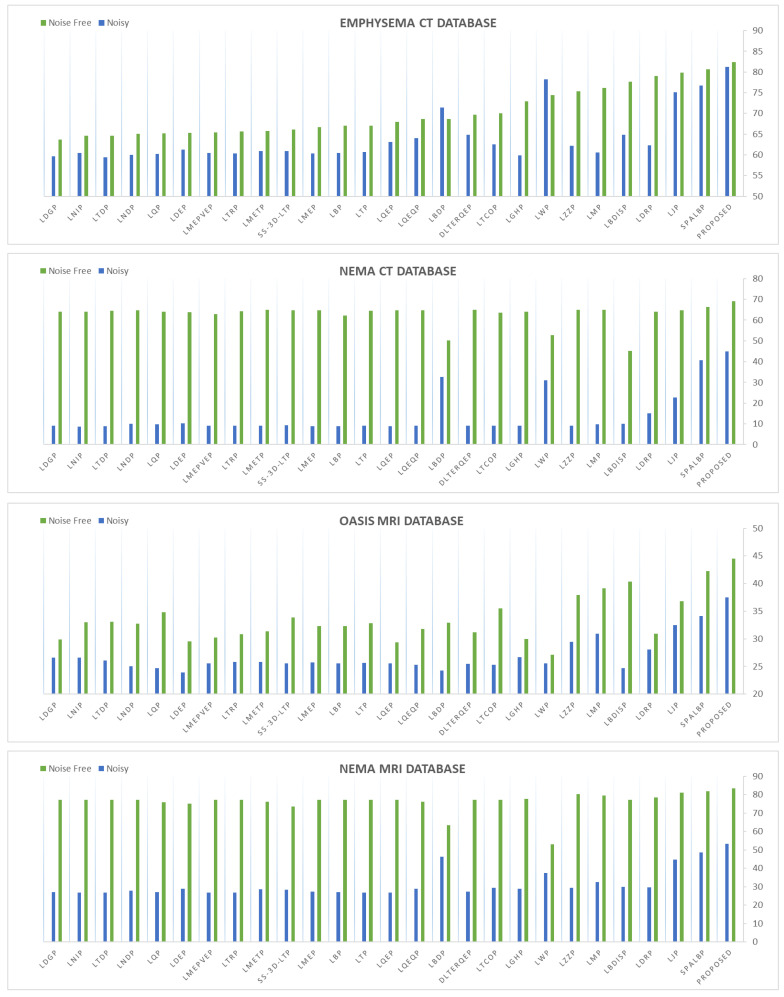
Proposed approach’s retrieval performance in comparison to all other methods in terms of avgP on noisy and noise-free images of four test datasets.

**Figure 9:**
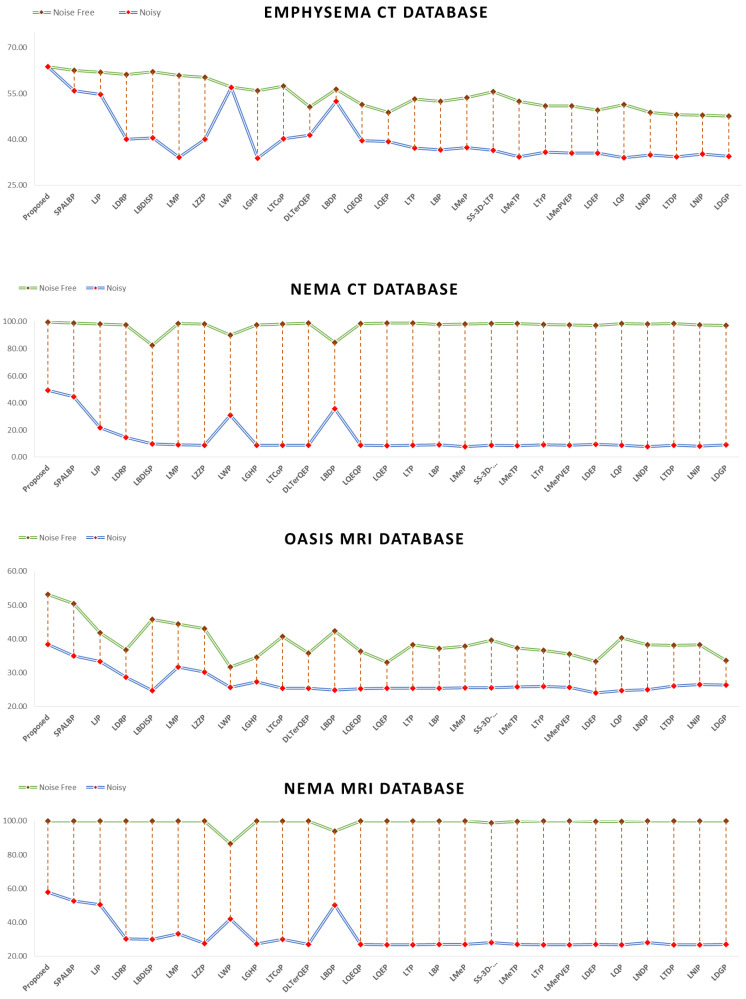
Proposed approach’s retrieval performance in comparison to all other methods in terms of MavgP on noisy and noise-free images of four test datasets.

**Figure 10:**
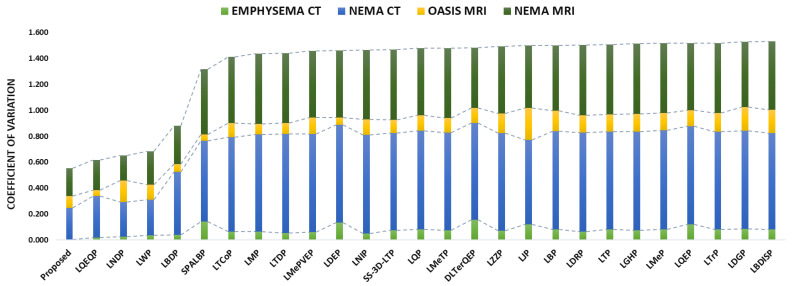
Proposed approach’s retrieval performance in comparison to all other methods in terms of CV (coefficient of variation) on noisy and noise-free images of four test datasets.

This section highlights the noise robustness of the proposed and all the compared approaches to retrieve images similar to that of a noise-degraded query image. Table 8, Table 9, Table 10 and Table 11 compare the performance of the proposed MsNrRiTxP approach with other methods on noise-induced versions of four test datasets. The proposed approach obtained retrieval rates of 81.28% (avgR), 46.95% (avgP), 59.52% ($F_{score}$), and 63.62% (MavgP) on noisy images from the Emphysema CT database; 64.78% (avgR), 44.78% (avgP), 52.95% ($F_{score}$), and 49.27% (MavgP) on noisy images from the NEMA CT database; 38.56% (avgR), 37.50% (avgP), 38.02% ($F_{score}$), and 38.44% (MavgP) on noisy images from the OASIS MRI database; and 69.92% (avgR), 53.36% (avgP), 60.53% ($F_{score}$), and 57.95% (MavgP) on noisy images from the NEMA MRI database; these are close to the 82.36% (avgR), 46.34% (avgP), 59.31% ($F_{score}$), and 63.82% (MavgP); 98.71% (avgR), 69.11% (avgP), 81.30% ($F_{score}$), and 99.56% (MavgP); 42.93% (avgR), 44.52% (avgP), 43.71% ($F_{score}$), and 53.11% (MavgP); and 100.00% (avgR), 83.47% (avgP), 90.99% ($F_{score}$), and 100.00% (MavgP) obtained on their noise-free variants, respectively. The noise-resilient characteristic demonstrated by the proposed approach allowed it to amass an average improvement in retrieval rates of 17.78% (avgR), 11.38% (avgP), 13.93% ($F_{score}$), and 24.03% (MavgP) on noisy images from the Emphysema CT database; 42.42% (avgR), 31.81% (avgP), 36.58% ($F_{score}$), and 36.51% (MavgP) on noisy images from the NEMA CT database; 12.84% (avgR), 10.95% (avgP), 11.90% ($F_{score}$), and 11.69% (MavgP) on noisy images from the OASIS MRI database; and 36.70% (avgR), 22.74% (avgP), 28.74% ($F_{score}$), and 26.93% (MavgP) on noisy images from the NEMA MRI database over all the compared approaches. The query results under noise conditions are shown in Figure 7. Figure 8 and Figure 9 present the comparative performance analysis of the proposed and the compared methods for noisy and noise-free images of all four test datasets in context with avgP and MavgP values, respectively. The figure clearly illustrates that all the approaches under review had a substantial decrease in their retrieval rates when tested on the four noisy databases. This decline, in comparison to noise-free images, underlines their inability to effectively retrieve images in noisy environments. The decrease in retrieval rates of the proposed approach is negligible in comparison to those of the existing approaches. The same can also be substantiated from Figure 10, which clearly depicts that the proposed approach yielded the minimum coefficient of variation (CV) among all the compared methods over noise-free and noisy images from all four test datasets. The formula for computing CV is given below. The lower values of CV signify that the proposed approach is highly robust to noise and its performance is least affected by the degradation. These results corroborate the effectiveness of the suggested approach in efficiently retrieving similar images, even in challenging conditions.

(46) $$CV=\frac{\mathrm{Standard}\mathrm{Deviation}}{\mathrm{Mean}}$$

One of the greatest strengths of the proposed MsNrRiTxP approach is its robustness to image noise, which means that even in situations where the query image suffers from noise degradation, the proposed approach is still able to extract the intricate texture and shape details buried within the noise. This allows our approach to generate excellent retrieval rates that are almost on par with those obtained on noise-free images. Among all the compared methods, the proposed method exhibited minimum deviation in retrieval rates on noisy and noise-free images, altogether attaining the highest retrieval performances on all four test datasets. This robustness of the proposed approach is attributed to the use of neutrosophic images for texture and shape extraction. The indeterminacy component in neutrosophic images helps in capturing and representing uncertain regions, which can arise due to noise or artifacts in the image. By incorporating indeterminacy, neutrosophic images tend to be more robust to noise compared to other approaches. Neutrosophic images provide a comprehensive representation of texture information by including truth (representing a certain or true texture), indeterminacy (capturing uncertain or ambiguous texture regions), and falsity (indicating false or non-texture regions). This comprehensive representation allows for a nuanced understanding of the texture patterns within the image.

## 5. Conclusions

In this paper, a new effective and robust descriptor, i.e., MsNrRiTxP, pattern has been presented to perform content-based retrieval of medical images in an attempt towards the development of computer-assisted diagnosis systems. The key contributions of the proposed work are the design of a novel pattern descriptor based in the neutrosophic domain, where, corresponding to every medical image, three neutrosophic images, i.e., truth (*T*), indeterminacy (*I*), and falsity (*F*) are obtained. These images provide a comprehensive representation of texture information by including truth (representing a certain or true texture), indeterminacy (capturing uncertain or ambiguous texture regions), and falsity (indicating false or non-texture regions). This comprehensive representation allows for a nuanced understanding of the texture patterns within the image. The MsNrRiTxP pattern is composed of three different patterns, i.e., MsTrP, NrTxP, and RiTxP, which extracts noise-resistant and rotation-invariant texture and shape features at multiple scales from each of the three neutrosophic images. The histogram of the proposed MsNrRiTxP pattern is generated by scale-wise concatenation of the joint histograms of MsTrP×RiTxP and NrTxP×RiTxP. The proposed method has been tested on both noisy and noise-free CT and MRI images from four standard test datasets. The experimental results confirm the superiority of the proposed pattern as compared to the existing state-of-the-art texture classifying descriptors. The average improvement in the retrieval rates achieved by the proposed approach over the compared approaches is very significant, especially in the case of noisy images. This substantiates the noise-robustness of the proposed approach, which is primarily achieved through infusion of neutrosophic information in the construction of MsNrRiTxP.

## Figures and Tables

**Table 1:** An illustration describing the count of elements in neighbor vector and median quantized neighbor vector at different scales.

Scale	*r*	$|p_{r}^{\left(i,j\right)}|$	$|{\mathrm{mqp}}_{r}^{\left(i,j\right)}|$
1	1	8	8
2	2	16	8 (Pair-wise median filtering)
3	3	24	8 (Triplet-wise median filtering)
4	4	32	8 (Quadruplet-wise median filtering)
5	5	40	8 (Quintuplet-wise median filtering)
6	6	48	8 (Sextuplet-wise median filtering)
7	7	56	8 (Septuplet-wise median filtering)
8	8	64	8 (Octuplet-wise median filtering)
9	9	72	8 (Nonuplet-wise median filtering)

**Table 2:** Names and abbreviations of all the compared methods.

S. No.	Abbreviation	Method Name
1	LBP	Local Binary Pattern
2	LTP	Local Ternary Pattern
3	LQP	Local Quinary Pattern
4	LTrP	Local Tetra Pattern
5	LTCoP	Local Ternary Co-Occurrence Pattern
6	LMeP	Local Mesh Pattern
7	LMePVEP	Local Mesh Peak Valley Edge Pattern
8	LDEP	Local Diagonal Extrema Pattern
9	LWP	Local Wavelet Pattern
10	LQEP	Local Quantized Extrema Pattern
11	SS-3D-LTP	Spherical Symmetric 3D Local Ternary Pattern
12	LBDP	Local Bit-Plane Decoded Pattern
13	LBDISP	Local Bit-Plane Dissimilarity Pattern
14	DLTerQEP	Directional Local Ternary Quantized Extrema Pattern
15	LTDP	Local Tri-Directional Pattern
16	LGHP	Local Gradient Hexa Pattern
17	LDGP	Local Directional Gradient Pattern
18	LQEQP	Local Quantized Extrema Quinary Pattern
19	LMeTP	Local Mesh Ternary Patterns
20	LNIP	Local Neighborhood Intensity Pattern
21	LNDP	Local Neighborhood Difference Pattern
22	LDZZP	Local Directional ZigZag Pattern
23	LMP	Local Morphological Pattern
24	LJP	Local Jet Pattern
25	LDRP	Local Directional Relation Pattern
26	SPALBP	Scale and Pattern Adaptive Local Binary Pattern

**Table 3:** Summary of datasets used in the experimental setup.

	CT Image Datasets		MR Image Datasets	
	**Emphysema CT**	**NEMA CT**	**OASIS MRI**	**NEMA MRI**
**No. of Images**	168	600	416	372
**Image Size**	61 × 61	512 × 512	208 × 208	256 × 256
**No. of Classes**	3	10	4	5
**Images per Class**	59, 50, 59	54, 70, 66, 50, 15	125, 104, 91, 96	72, 100, 76, 59, 65
		60, 52, 104, 60, 69		

**Table 4:** Table representing the proposed approach’s retrieval performance in comparison to all other methods on Emphysema CT database. The values are expressed as percentages (%).

	Performance Measures				Improvement			
	***avgR***	***avgP***	$F_{\mathrm{score}}$	***MavgP***	**(Proposed–Compared)**			
**Proposed**	**82.36**	**46.34**	**59.31**	**63.82**	***avgR***	***avgP***	$F_{\mathrm{score}}$	***MavgP***
SPALBP	80.71	45.41	58.12	62.54	1.65	0.93	1.19	1.28
LJP	79.89	44.95	57.53	61.91	2.47	1.39	1.78	1.91
LDRP	79.06	44.49	56.94	61.27	3.30	1.85	2.37	2.55
LBDISP	77.69	43.48	55.75	62.20	4.67	2.86	3.56	1.62
LMP	76.14	42.61	54.64	60.96	6.22	3.73	4.67	2.86
LZZP	75.36	42.18	54.08	60.33	7.00	4.16	5.23	3.49
LWP	74.46	41.35	53.17	57.08	7.90	4.99	6.14	6.74
LGHP	72.97	40.52	52.11	55.94	9.39	5.82	7.20	7.88
LTCoP	70.08	39.15	50.24	57.39	12.28	7.19	9.07	6.43
DLTerQEP	69.64	39.63	50.51	50.72	12.72	6.71	8.80	13.10
LBDP	68.69	38.04	48.97	56.41	13.67	8.30	10.34	7.41
LQEQP	68.67	38.98	49.73	51.44	13.69	7.36	9.58	12.38
LQEP	68.00	38.32	49.02	48.91	14.36	8.02	10.29	14.91
LTP	66.99	37.53	48.11	53.20	15.37	8.81	11.20	10.62
LBP	66.98	37.53	48.11	52.41	15.38	8.81	11.20	11.41
LMeP	66.68	37.27	47.81	53.72	15.68	9.07	11.50	10.10
SS-3D-LTP	66.12	36.98	47.43	55.61	16.24	9.36	11.88	8.21
LMeTP	65.82	36.83	47.23	52.41	16.54	9.51	12.08	11.41
LTrP	65.61	36.86	47.20	50.95	16.75	9.48	12.11	12.87
LMePVEP	65.45	36.70	47.03	50.96	16.91	9.64	12.28	12.86
LDEP	65.34	36.56	46.89	49.61	17.02	9.78	12.42	14.21
LQP	65.21	36.51	46.81	51.47	17.15	9.83	12.50	12.35
LNDP	65.10	36.52	46.80	48.86	17.26	9.82	12.51	14.96
LTDP	64.62	36.16	46.37	48.02	17.74	10.18	12.94	15.80
LNIP	64.61	36.20	46.40	47.99	17.75	10.14	12.91	15.83
LDGP	63.64	35.55	45.61	47.57	18.72	10.79	13.70	16.25
**Average Improvement**					**12.61**	**7.25**	**9.21**	**9.59**

**Table 5:** Table representing the proposed approach’s retrieval performance in comparison to all other methods on the NEMA CT database. The values are expressed as percentages (%).

	Performance Measures				Improvement			
	***avgR***	***avgP***	$F_{\mathrm{score}}$	***MavgP***	**(Proposed–Compared)**			
**Proposed**	**98.71**	**69.11**	**81.30**	**99.56**	***avgR***	***avgP***	$F_{\mathrm{score}}$	***MavgP***
SPALBP	97.72	66.35	79.03	99.06	0.99	2.76	2.27	0.50
DLTerQEP	96.45	64.92	77.61	99.00	2.26	4.19	3.69	0.56
LQEP	96.43	64.70	77.44	98.96	2.28	4.41	3.86	0.60
LNDP	96.42	64.62	77.38	98.07	2.29	4.49	3.92	1.49
LMeTP	96.36	64.98	77.62	98.55	2.35	4.13	3.68	1.01
LMP	96.26	64.92	77.54	98.45	2.45	4.19	3.76	1.11
LZZP	96.17	64.85	77.46	98.35	2.54	4.26	3.84	1.21
LJP	96.07	64.79	77.39	98.25	2.64	4.32	3.91	1.31
LMeP	96.19	64.64	77.32	98.12	2.52	4.47	3.98	1.44
LTDP	96.18	64.50	77.21	98.50	2.53	4.61	4.09	1.06
LTP	95.99	64.52	77.17	98.83	2.72	4.59	4.13	0.73
SS-3D-LTP	95.92	64.62	77.22	98.43	2.79	4.49	4.08	1.13
LTrP	95.92	64.26	76.96	97.92	2.79	4.85	4.34	1.64
LQEQP	95.83	64.65	77.21	98.56	2.88	4.46	4.09	1.00
LDRP	95.71	64.11	76.78	97.46	3.00	5.00	4.51	2.10
LGHP	95.62	64.04	76.71	97.37	3.09	5.07	4.59	2.19
LDGP	95.52	63.98	76.63	97.27	3.19	5.13	4.67	2.29
LTCoP	95.47	63.55	76.31	98.36	3.24	5.56	4.99	1.20
LNIP	95.45	63.93	76.57	97.68	3.26	5.18	4.73	1.88
LQP	95.27	64.00	76.56	98.48	3.44	5.11	4.74	1.08
LDEP	95.02	63.72	76.28	97.23	3.69	5.39	5.02	2.33
LBP	94.19	62.25	74.96	97.85	4.52	6.86	6.34	1.71
LMePVEP	93.51	62.91	75.22	97.43	5.20	6.20	6.08	2.13
LWP	80.37	52.76	63.70	89.89	18.34	16.35	17.60	9.67
LBDP	76.91	50.06	60.64	84.45	21.80	19.05	20.66	15.11
LBDISP	71.14	45.20	55.28	82.41	27.57	23.91	26.02	17.15
**Average Improvement**					**5.17**	**6.50**	**6.29**	**2.83**

**Table 6:** Table representing the proposed approach’s retrieval performance in comparison to all other methods on OASIS MRI database. The values are expressed as percentages (%).

	Performance Measures				Improvement			
	***avgR***	***avgP***	$F_{\mathrm{score}}$	***MavgP***	**(Proposed–Compared)**			
**Proposed**	**42.93**	**44.52**	**43.71**	**53.11**	***avgR***	***avgP***	$F_{\mathrm{score}}$	***MavgP***
SPALBP	40.78	42.29	41.53	50.45	2.15	2.23	2.19	2.66
LBDISP	37.89	40.35	39.08	45.81	5.04	4.17	4.63	7.30
LMP	36.75	39.14	37.91	44.44	6.18	5.38	5.80	8.67
LZZP	35.65	37.97	36.77	43.10	7.28	6.55	6.94	10.01
LJP	34.58	36.83	35.67	41.81	8.35	7.69	8.04	11.30
LTCoP	33.30	35.51	34.37	40.73	9.63	9.01	9.34	12.38
LQP	32.69	34.78	33.70	40.26	10.24	9.74	10.01	12.85
SS-3D-LTP	31.84	33.84	32.81	39.58	11.09	10.68	10.90	13.53
LBDP	31.18	32.87	32.01	42.40	11.75	11.65	11.70	10.71
LTDP	30.99	33.12	32.02	38.17	11.94	11.40	11.69	14.94
LNIP	30.88	33.00	31.90	38.29	12.05	11.52	11.81	14.82
LTP	30.76	32.79	31.74	38.20	12.17	11.73	11.97	14.91
LNDP	30.75	32.75	31.72	38.30	12.18	11.77	11.99	14.81
LBP	30.29	32.26	31.25	37.13	12.64	12.26	12.46	15.98
LMeP	30.26	32.29	31.24	37.84	12.67	12.23	12.47	15.27
LQEQP	29.94	31.74	30.82	36.37	12.99	12.78	12.89	16.74
LMeTP	29.46	31.37	30.39	37.32	13.47	13.15	13.32	15.79
DLTerQEP	29.43	31.15	30.27	35.87	13.50	13.37	13.44	17.24
LDRP	29.13	30.93	30.00	36.77	13.80	13.59	13.71	16.34
LTrP	29.04	30.84	29.92	36.66	13.89	13.68	13.79	16.45
LMePVEP	28.33	30.19	29.23	35.49	14.60	14.33	14.48	17.62
LGHP	28.11	29.95	29.00	34.61	14.82	14.57	14.71	18.50
LDGP	28.08	29.92	28.97	33.60	14.85	14.60	14.74	19.51
LDEP	27.84	29.51	28.65	33.39	15.09	15.01	15.06	19.72
LQEP	27.61	29.38	28.47	33.13	15.32	15.14	15.24	19.98
LWP	25.65	27.12	26.36	31.66	17.28	17.40	17.35	21.45
**Average Improvement**					**11.73**	**11.37**	**11.56**	**14.60**

**Table 7:** Table representing the proposed approach’s retrieval performance in comparison to all other methods on NEMA MRI database. The values are expressed as percentages (%).

	Performance Measures				Improvement			
	***avgR***	***avgP***	$F_{\mathrm{score}}$	***MavgP***	**(Proposed–Compared)**			
**Proposed**	**100.00**	**83.47**	**90.99**	**100.00**	***avgR***	***avgP***	$F_{\mathrm{score}}$	***MavgP***
SPALBP	100.00	81.80	89.99	100.00	0.00	1.67	1.00	0.00
LJP	100.00	80.98	89.49	100.00	0.00	2.49	1.50	0.00
LZZP	100.00	80.17	89.00	100.00	0.00	3.30	1.99	0.00
LMP	100.00	79.37	88.50	100.00	0.00	4.10	2.49	0.00
LDRP	100.00	78.58	88.00	100.00	0.00	4.89	2.99	0.00
LGHP	100.00	77.79	87.51	100.00	0.00	5.68	3.48	0.00
LBP	100.00	77.06	87.04	100.00	0.00	6.41	3.95	0.00
LTP	100.00	77.06	87.04	100.00	0.00	6.41	3.95	0.00
LTrP	100.00	77.06	87.04	100.00	0.00	6.41	3.95	0.00
LTCoP	100.00	77.06	87.04	100.00	0.00	6.41	3.95	0.00
LMeP	100.00	77.06	87.04	100.00	0.00	6.41	3.95	0.00
LMePVEP	100.00	77.06	87.04	100.00	0.00	6.41	3.95	0.00
LNIP	100.00	77.06	87.04	100.00	0.00	6.41	3.95	0.00
LTDP	100.00	77.06	87.04	100.00	0.00	6.41	3.95	0.00
LNDP	100.00	77.06	87.04	100.00	0.00	6.41	3.95	0.00
LDGP	100.00	77.06	87.04	100.00	0.00	6.41	3.95	0.00
LQEP	100.00	77.06	87.04	100.00	0.00	6.41	3.95	0.00
DLTerQEP	100.00	77.06	87.04	100.00	0.00	6.41	3.95	0.00
LBDISP	99.99	77.05	87.03	99.99	0.01	6.42	3.96	0.01
LQEQP	98.83	76.06	85.96	99.86	1.17	7.41	5.03	0.14
LMeTP	98.80	76.06	85.95	99.77	1.20	7.41	5.04	0.23
LQP	98.79	75.95	85.88	99.80	1.21	7.52	5.11	0.20
LDEP	97.90	74.96	84.91	99.63	2.10	8.51	6.08	0.37
SS-3D-LTP	96.22	73.51	83.34	98.87	3.78	9.96	7.65	1.13
LBDP	83.80	63.36	72.16	93.93	16.20	20.11	18.83	6.07
LWP	71.51	53.04	60.91	86.68	28.49	30.43	30.08	13.32
**Average Improvement**					**2.08**	**7.57**	**5.49**	**0.83**

**Table 8:** Table representing the proposed approach’s retrieval performance in comparison to all other methods on noisy images of Emphysema CT database. The values are expressed as percentages (%).

	Performance Measures				Improvement			
	***avgR***	***avgP***	$F_{\mathrm{score}}$	***MavgP***	**(Proposed–Compared)**			
**Proposed**	**81.28**	**46.95**	**59.52**	**63.72**	***avgR***	***avgP***	$F_{\mathrm{score}}$	***MavgP***
LWP	78.26	43.63	56.03	57.06	3.02	3.32	3.49	6.66
SPALBP	76.69	42.76	54.91	55.92	4.59	4.19	4.61	7.80
LJP	75.16	41.90	53.81	54.80	6.12	5.05	5.71	8.92
LBDP	71.41	39.64	50.98	52.41	9.87	7.31	8.54	11.31
DLTerQEP	64.85	37.02	47.13	41.42	16.43	9.93	12.39	22.30
LBDISP	64.86	36.47	46.69	40.55	16.42	10.48	12.83	23.17
LQEQP	64.08	36.05	46.14	39.71	17.20	10.90	13.38	24.01
LQEP	63.15	35.64	45.56	39.32	18.13	11.31	13.95	24.40
LTCoP	62.51	35.02	44.89	40.27	18.77	11.93	14.63	23.45
LDRP	62.32	34.91	44.76	40.15	18.96	12.04	14.76	23.57
LZZP	62.14	34.81	44.62	40.03	19.14	12.14	14.90	23.69
LDEP	61.22	34.68	44.28	35.60	20.06	12.27	15.24	28.12
LBP	60.51	34.08	43.60	36.55	20.77	12.87	15.92	27.17
LTP	60.72	34.06	43.64	37.24	20.56	12.89	15.88	26.48
LMeP	60.35	33.94	43.45	37.43	20.93	13.01	16.07	26.29
LMePVEP	60.50	33.93	43.48	35.52	20.78	13.02	16.04	28.20
LTrP	60.30	33.92	43.42	35.86	20.98	13.03	16.10	27.86
SS-3D-LTP	60.90	33.90	43.56	36.48	20.38	13.05	15.96	27.24
LNIP	60.42	33.84	43.38	35.22	20.86	13.11	16.14	28.50
LMeTP	60.90	33.84	43.51	34.33	20.38	13.11	16.01	29.39
LMP	60.60	33.67	43.29	34.16	20.68	13.28	16.23	29.56
LNDP	59.96	33.61	43.07	34.95	21.32	13.34	16.44	28.77
LQP	60.22	33.44	43.00	34.08	21.06	13.51	16.52	29.64
LGHP	59.92	33.43	42.92	33.91	21.36	13.52	16.60	29.81
LDGP	59.67	33.43	42.85	34.57	21.61	13.52	16.67	29.15
LTDP	59.38	33.07	42.48	34.36	21.90	13.88	17.04	29.36
**Average Improvement**					**17.78**	**11.38**	**13.93**	**24.03**

**Table 9:** Table representing the proposed approach’s retrieval performance in comparison to all other methods on noisy images of NEMA CT database. The values are expressed as percentages (%).

	Performance Measures				Improvement			
	***avgR***	***avgP***	$F_{\mathrm{score}}$	***MavgP***	**(Proposed–Compared)**			
**Proposed**	**64.78**	**44.78**	**52.95**	**49.27**	***avgR***	***avgP***	$F_{\mathrm{score}}$	***MavgP***
SPALBP	58.89	40.71	48.14	44.79	5.89	4.07	4.81	4.48
LBDP	47.11	32.57	38.51	35.83	17.67	12.21	14.44	13.44
LWP	46.51	31.03	37.23	31.00	18.27	13.75	15.72	18.27
LJP	42.86	22.75	29.72	21.85	21.91	22.04	23.23	27.42
LDRP	28.58	15.17	19.81	14.57	36.20	29.62	33.14	34.70
LBDISP	19.05	10.11	13.21	9.71	45.73	34.67	39.74	39.56
LQP	18.45	9.73	12.74	8.83	46.33	35.05	40.21	40.44
LDEP	18.39	10.27	13.18	9.48	46.39	34.51	39.77	39.79
LMP	17.47	9.76	12.52	9.01	47.31	35.03	40.43	40.26
LDGP	17.28	9.05	11.88	8.99	47.50	35.73	41.07	40.28
LTrP	17.03	9.16	11.91	9.03	47.75	35.62	41.04	40.24
LMePVEP	16.81	9.18	11.87	8.69	47.97	35.60	41.08	40.58
LTP	16.80	9.18	11.87	8.87	47.98	35.60	41.08	40.40
LZZP	16.80	9.18	11.87	8.87	47.98	35.60	41.08	40.40
LTCoP	16.77	9.14	11.83	8.72	48.01	35.64	41.12	40.55
SS-3D-LTP	16.75	9.24	11.91	8.77	48.03	35.54	41.04	40.50
DLTerQEP	16.68	9.10	11.77	8.66	48.10	35.68	41.18	40.61
LMeTP	16.67	9.18	11.84	8.61	48.11	35.60	41.11	40.66
LQEQP	16.67	9.18	11.84	8.68	48.11	35.60	41.11	40.59
LGHP	16.67	9.18	11.84	8.68	48.11	35.60	41.11	40.59
LMeP	16.66	8.91	11.61	7.67	48.12	35.87	41.34	41.60
LTDP	16.59	8.97	11.64	8.70	48.19	35.81	41.31	40.57
LBP	16.59	8.79	11.49	9.22	48.19	35.99	41.46	40.05
LQEP	16.55	8.93	11.60	8.52	48.23	35.85	41.35	40.75
LNDP	16.42	10.09	12.50	7.88	48.36	34.69	40.45	41.39
LNIP	16.24	8.67	11.31	8.01	48.54	36.11	41.64	41.26
**Average Improvement**					**42.42**	**31.81**	**36.58**	**36.51**

**Table 10:** Table representing the proposed approach’s retrieval performance in comparison to all other methods on noisy images of OASIS MRI database. The values are expressed as percentages (%).

	Performance Measures				Improvement			
	***avgR***	***avgP***	$F_{\mathrm{score}}$	***MavgP***	**(Proposed–Compared)**			
**Proposed**	**38.56**	**37.50**	**38.02**	**38.44**	***avgR***	***avgP***	$F_{\mathrm{score}}$	***MavgP***
SPALBP	35.06	34.09	34.56	34.94	3.51	3.41	3.46	3.49
LJT	33.39	32.46	32.92	33.28	5.17	5.03	5.10	5.16
LMP	31.80	30.92	31.35	31.69	6.76	6.58	6.67	6.74
LZZP	30.28	29.45	29.86	30.19	8.28	8.05	8.16	8.25
LDRP	28.84	28.04	28.44	28.75	9.72	9.45	9.58	9.69
LGHP	27.47	26.71	27.08	27.38	11.09	10.79	10.94	11.06
LBDP	24.97	24.28	24.62	24.89	13.59	13.22	13.40	13.55
LWP	24.47	25.56	25.00	25.76	14.09	11.94	13.02	12.68
LBDISP	24.31	24.72	24.51	24.75	14.25	12.78	13.51	13.69
SS-3D-LTP	24.17	25.54	24.84	25.55	14.39	11.96	13.18	12.89
LTP	24.15	25.60	24.85	25.40	14.41	11.90	13.17	13.04
LDGP	24.11	26.60	25.30	26.35	14.45	10.90	12.72	12.09
LMePVEP	24.07	25.55	24.79	25.70	14.49	11.95	13.23	12.74
LTDP	24.06	26.06	25.02	26.08	14.50	11.44	13.00	12.36
LQEP	24.05	25.55	24.78	25.43	14.51	11.95	13.24	13.01
LTrP	24.04	25.84	24.91	25.91	14.52	11.66	13.11	12.53
LDEP	24.03	23.89	23.96	24.02	14.53	13.61	14.06	14.42
LQP	24.02	24.67	24.34	24.80	14.54	12.83	13.68	13.64
LNIP	24.01	26.55	25.21	26.47	14.55	10.95	12.81	11.97
LBP	24.01	25.59	24.78	25.49	14.55	11.91	13.24	12.95
LMeTP	24.00	25.77	24.85	25.81	14.56	11.73	13.17	12.63
LMeP	23.95	25.70	24.80	25.62	14.61	11.80	13.22	12.82
DLTerQEP	23.95	25.50	24.70	25.41	14.61	12.00	13.32	13.03
LQEQP	23.95	25.32	24.62	25.24	14.61	12.18	13.40	13.20
LTCoP	23.90	25.27	24.57	25.37	14.66	12.23	13.45	13.07
LNDP	23.78	24.99	24.37	25.07	14.78	12.51	13.65	13.37
**Average Improvement**					**12.84**	**10.95**	**11.90**	**11.69**

**Table 11:** Table representing the proposed approach’s retrieval performance in comparison to all other methods on noisy images of NEMA MRI database. The values are expressed as percentages (%).

	Performance Measures				Improvement			
	***avgR***	***avgP***	$F_{\mathrm{score}}$	***MavgP***	**(Proposed–Compared)**			
**Proposed**	**69.92**	**53.36**	**60.53**	**57.95**	***avgR***	***avgP***	$F_{\mathrm{score}}$	***MavgP***
SPALBP	63.57	48.51	55.03	52.68	6.36	4.85	5.50	5.27
LBDP	60.54	46.20	52.40	50.17	9.38	7.16	8.13	7.78
LJP	55.61	44.80	49.62	50.45	14.32	8.57	10.91	7.50
LWP	46.34	37.33	41.35	42.04	23.58	16.03	19.18	15.91
LBDISP	34.91	29.98	32.26	30.05	35.01	23.38	28.27	27.90
LMP	33.73	32.59	33.15	33.24	36.19	20.78	27.38	24.71
LDRP	30.66	29.62	30.13	30.22	39.26	23.74	30.40	27.73
LTCoP	30.36	29.33	29.84	29.92	39.56	24.03	30.69	28.03
LZZP	30.49	29.26	29.87	27.45	39.43	24.10	30.66	30.50
LGHP	30.19	28.97	29.57	27.18	39.73	24.39	30.96	30.77
LQEQP	30.04	28.83	29.42	27.04	39.88	24.53	31.11	30.91
LNDP	29.90	27.92	28.88	28.14	40.02	25.44	31.65	29.81
LDEP	29.84	28.96	29.39	27.08	40.08	24.40	31.14	30.87
SS-3D-LTP	29.56	28.28	28.91	28.06	40.36	25.08	31.62	29.89
LMeTP	29.45	28.69	29.06	26.90	40.47	24.67	31.47	31.05
LQP	28.36	27.05	27.69	26.63	41.56	26.31	32.84	31.32
LMeP	27.37	27.20	27.29	26.92	42.55	26.16	33.24	31.03
DLTerQEP	27.33	27.20	27.26	26.92	42.59	26.16	33.27	31.03
LBP	27.28	27.14	27.21	27.14	42.64	26.22	33.32	30.81
LDGP	26.96	26.94	26.95	26.95	42.96	26.42	33.58	31.00
LMePVEP	26.90	26.89	26.90	26.88	43.02	26.47	33.63	31.07
LTP	26.89	26.89	26.89	26.88	43.03	26.47	33.64	31.07
LNIP	26.89	26.88	26.88	26.88	43.03	26.48	33.65	31.07
LTDP	26.89	26.88	26.89	26.88	43.03	26.48	33.64	31.07
LTrP	26.88	26.88	26.88	26.88	43.04	26.48	33.65	31.07
LQEP	26.88	26.88	26.88	26.88	43.04	26.48	33.65	31.07
**Average Improvement**					**36.70**	**22.74**	**28.74**	**26.93**

## Data Availability

The data presented in this study will be made available on reasonable request to the corresponding author.

## References

[1] Webb A. (2022). Introduction to Biomedical Imaging.

[2] Nishikawa R.M. (2010). Computer-aided detection and diagnosis. Digital Mammography.

[3] Ghosh P., Antani S., Long L.R., Thoma G.R. Review of medical image retrieval systems and future directions. Proceedings of the 2011 24th International Symposium on Computer-Based Medical Systems (CBMS).

[4] Kumar A., Kim J., Cai W., Fulham M., Feng D. (2013). Content-based medical image retrieval: A survey of applications to multidimensional and multimodality data. J. Digit. Imaging.

[5] Cai W., Song Y., Kumar A., Kim J., Feng D.D. (2020). Content-based large-scale medical image retrieval. Biomedical Information Technology.

[6] Rui Y., Huang T.S., Chang S.F. (1999). Image retrieval: Current techniques, promising directions, and open issues. J. Vis. Commun. Image Represent..

[7] Banuchitra S., Kungumaraj K. (2016). A comprehensive survey of content based image retrieval techniques. Int. J. Eng. Comput. Sci..

[8] Nixon M., Aguado A. (2019). Feature Extraction and Image Processing for Computer Vision.

[9] Ping Tian D. (2013). A review on image feature extraction and representation techniques. Int. J. Multimed. Ubiquitous Eng..

[10] Ojala T., Pietikäinen M., Harwood D. (1996). A comparative study of texture measures with classification based on featured distributions. Pattern Recognit..

[11] Nanni L., Lumini A., Brahnam S. (2010). Local binary patterns variants as texture descriptors for medical image analysis. Artif. Intell. Med..

[12] Tan X., Triggs B. (2010). Enhanced local texture feature sets for face recognition under difficult lighting conditions. IEEE Trans. Image Process..

[13] Murala S., Wu Q.J. (2013). Local ternary co-occurrence patterns: A new feature descriptor for MRI and CT image retrieval. Neurocomputing.

[14] Murala S., Wu Q.J. (2013). Local mesh patterns versus local binary patterns: Biomedical image indexing and retrieval. IEEE J. Biomed. Health Inform..

[15] Murala S., Wu Q.J. (2014). MRI and CT image indexing and retrieval using local mesh peak valley edge patterns. Signal Process. Image Commun..

[16] Murala S., Wu Q.J. (2015). Spherical symmetric 3D local ternary patterns for natural, texture and biomedical image indexing and retrieval. Neurocomputing.

[17] Dubey S.R., Singh S.K., Singh R.K. (2015). Local wavelet pattern: A new feature descriptor for image retrieval in medical CT databases. IEEE Trans. Image Process..

[18] Dubey S.R., Singh S.K., Singh R.K. (2015). Local diagonal extrema pattern: A new and efficient feature descriptor for CT image retrieval. IEEE Signal Process. Lett..

[19] Dubey S.R., Singh S.K., Singh R.K. (2016). Novel local bit-plane dissimilarity pattern for computed tomography image retrieval. Electron. Lett..

[20] Dubey S.R., Singh S.K., Singh R.K. (2015). Local bit-plane decoded pattern: A novel feature descriptor for biomedical image retrieval. IEEE J. Biomed. Health Inform..

[21] Deep G., Kaur L., Gupta S. (2018). Local mesh ternary patterns: A new descriptor for MRI and CT biomedical image indexing and retrieval. Comput. Methods Biomech. Biomed. Eng. Imaging Vis..

[22] Deep G., Kaur L., Gupta S. (2016). Directional local ternary quantized extrema pattern: A new descriptor for biomedical image indexing and retrieval. Eng. Sci. Technol. Int. J..

[23] Deep G., Kaur L., Gupta S. (2018). Local quantized extrema quinary pattern: A new descriptor for biomedical image indexing and retrieval. Comput. Methods Biomech. Biomed. Eng. Imaging Vis..

[24] Murala S., Maheshwari R.P., Balasubramanian R. (2012). Local tetra patterns: A new feature descriptor for content-based image retrieval. IEEE Trans. Image Process..

[25] Chakraborty S., Singh S.K., Chakraborty P. (2016). Local gradient hexa pattern: A descriptor for face recognition and retrieval. IEEE Trans. Circuits Syst. Video Technol..

[26] Verma M., Raman B. (2016). Local tri-directional patterns: A new texture feature descriptor for image retrieval. Digit. Signal Process..

[27] Verma M., Raman B. (2018). Local neighborhood difference pattern: A new feature descriptor for natural and texture image retrieval. Multimed. Tools Appl..

[28] Banerjee P., Bhunia A.K., Bhattacharyya A., Roy P.P., Murala S. (2018). Local neighborhood intensity pattern—A new texture feature descriptor for image retrieval. Expert Syst. Appl..

[29] Chakraborty S., Singh S.K., Chakraborty P. (2017). Local directional gradient pattern: A local descriptor for face recognition. Multimed. Tools Appl..

[30] Dubey S.R. (2019). Local directional relation pattern for unconstrained and robust face retrieval. Multimed. Tools Appl..

[31] Roy S.K., Chanda B., Chaudhuri B.B., Banerjee S., Ghosh D.K., Dubey S.R. (2018). Local directional ZigZag pattern: A rotation invariant descriptor for texture classification. Pattern Recognit. Lett..

[32] Roy S.K., Chanda B., Chaudhuri B.B., Ghosh D.K., Dubey S.R. (2020). Local jet pattern: A robust descriptor for texture classification. Multimed. Tools Appl..

[33] Roy S.K., Chanda B., Chaudhuri B.B., Ghosh D.K., Dubey S.R. (2018). Local morphological pattern: A scale space shape descriptor for texture classification. Digit. Signal Process..

[34] Agarwal M., Maheshwari R.P. (2020). Multichannel local ternary co-occurrence pattern for content-based image retrieval. Iran. J. Sci. Technol. Trans. Electr. Eng..

[35] Hu S., Li J., Fan H., Lan S., Pan Z. (2024). Scale and pattern adaptive local binary pattern for texture classification. Expert Syst. Appl..

[36] Qayyum A., Anwar S.M., Awais M., Majid M. (2017). Medical image retrieval using deep convolutional neural network. Neurocomputing.

[37] Swati Z.N.K., Zhao Q., Kabir M., Ali F., Ali Z., Ahmed S., Lu J. (2019). Content-based brain tumor retrieval for MR images using transfer learning. IEEE Access.

[38] Sudhish D.K., Nair L.R., Shailesh S. (2024). Content-based image retrieval for medical diagnosis using fuzzy clustering and deep learning. Biomed. Signal Process. Control.

[39] Lundervold A.S., Lundervold A. (2019). An overview of deep learning in medical imaging focusing on MRI. Z. Med. Phys..

[40] Anwar S.M., Majid M., Qayyum A., Awais M., Alnowami M., Khan M.K. (2018). Medical image analysis using convolutional neural networks: A review. J. Med. Syst..

[41] Broumi S., Bakali A., Bahnasse A. (2018). Neutrosophic sets: An overview. New Trends in Neutrosophic Theory and Applications.

[42] El-Hefenawy N., Metwally M.A., Ahmed Z.M., El-Henawy I.M. (2016). A review on the applications of neutrosophic sets. J. Comput. Theor. Nanosci..

[43] Salama A.A., Smarandache F., Eisa M. (2014). Introduction to image processing via neutrosophic techniques. Neutrosophic Sets Syst..

[44] Talouki A.G., Koochari A., Edalatpanah S.A. (2024). Image completion based on segmentation using neutrosophic sets. Expert Syst. Appl..

[45] Alsattar H.A., Qahtan S., Zaidan A.A., Deveci M., Martinez L., Pamucar D., Pedrycz W. (2024). Developing deep transfer and machine learning models of chest X-ray for diagnosing COVID-19 cases using probabilistic single-valued neutrosophic hesitant fuzzy. Expert Syst. Appl..

[46] Ojala T., Pietikainen M., Maenpaa T. (2002). Multiresolution gray-scale and rotation invariant texture classification with local binary patterns. IEEE Trans. Pattern Anal. Mach. Intell..

[47] Aswini K.R.N., Prakash S.P., Ravindran G., Jagadesh T., Naik A.V. An Extended Canberra Similarity Measure Method for Content-Based Image Retrieval. Proceedings of the 2023 International Conference on Evolutionary Algorithms and Soft Computing Techniques (EASCT).

[48] Emphysema-CT Database. http://image.diku.dk/emphysema_database/.

[49] OASIS-MRI Database. http://www.oasis-brains.org/.

